# Negative regulation of pro-apoptotic AMPK/JNK pathway by itaconate in mice with fulminant liver injury

**DOI:** 10.1038/s41419-023-06001-w

**Published:** 2023-07-31

**Authors:** Kerui Fan, Kun Chen, Xinyan Zan, Ying Zhi, Xue Zhang, Xinyue Zhang, Jinghuan Qiu, Gang Liu, Longjiang Li, Li Tang, Kai Hu, Jingyuan Wan, Xianqiong Gong, Yongqiang Yang, Li Zhang

**Affiliations:** 1 Department of Pathophysiology, Basic Medical College, Chongqing Medical University, Chongqing, China; 2 Laboratory of Stem Cell and Tissue Engineering, Chongqing Medical University, Chongqing, China; 3 Department of Emergency, University-Town Hospital of Chongqing Medical University, 401331 Chongqing, China; 4 Department of Pharmacology, Chongqing Medical University, Chongqing, China; 5 Hepatology Center, Xiamen Hospital of Traditional Chinese Medicine, Xiamen, Fujian Province China

**Keywords:** Apoptosis, Cell signalling

## Abstract

Accumulating evidence indicates that metabolic responses are deeply integrated into signal transduction, which provides novel opportunities for the metabolic control of various disorders. Recent studies suggest that itaconate, a highly concerned bioactive metabolite catalyzed by immune responsive gene 1 (IRG1), is profoundly involved in the regulation of apoptosis, but the underlying mechanisms have not been fully understood. In the present study, the molecular mechanisms responsible for the apoptosis-modulatory activities of IRG1/itaconate have been investigated in mice with lipopolysaccharide (LPS)/D-galactosamine (D-Gal)-induced apoptotic liver injury. The results indicated that LPS/D-Gal exposure upregulated the level of IRG1 and itaconate. Deletion of IRG1 resulted in exacerbated hepatocytes apoptosis and liver injury. The phospho-antibody microarray analysis and immunoblot analysis indicated that IRG1 deletion enhanced the activation of AMP-activated protein kinase (AMPK)/c-jun-N-terminal kinase (JNK) pathway in LPS/D-Gal exposed mice. Mechanistically, IRG1 deficiency impaired the anti-oxidative nuclear factor erythroid-2 related factor 2 (Nrf2) signaling and then enhanced the activation of the redox-sensitive AMPK/JNK pathway that promotes hepatocytes apoptosis. Importantly, post-insult supplementation with 4-octyl itaconate (4-OI), a cell-permeable derivate of itaconate, resulted in beneficial outcomes in fulminant liver injury. Therefore, IRG1/itaconate might function as a negative regulator that controls AMPK-induced hepatocyte apoptosis in LPS/D-Gal-induced fulminant liver injury.

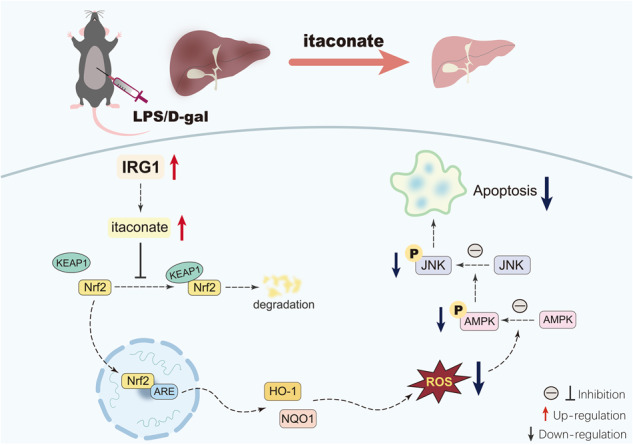

## Introduction

The uncontrolled hepatocyte apoptosis is a crucial pathological event involved in the development of various hepatic disorders [1, 2]. The fate of the hepatocytes is tightly regulated by a series of pro-apoptotic and anti-apoptotic signal pathways, which have been extensively investigated [3, 4]. Recently, a growing number of evidence indicates that the metabolic responses are deeply integrated into the regulation of apoptosis as well as other cellular events [5–7], which might provide new opportunities for the pharmacological intervention of hepatocytes apoptosis in hepatic disorders [8, 9].

In addition to energy supply and chemical support, metabolic responses also generate a lot of bioactive metabolites that are actively involved in signal transduction [10]. Itaconate is a by-product of the Krebs cycle, which is catalyzed by aconitate decarboxylase, also known as immune responsive gene 1 (IRG1) [11, 12]. Increasing evidence suggests that itaconate modulates several signal pathways involved in oxidative stress and inflammatory response [13–15]. In addition, itaconate is profoundly involved in the regulation of apoptosis in osteoblasts, neuronal cells, and endothelial cells [16–18], but the underlying mechanisms have not been fully elucidated.

Massive hepatocytes apoptosis is a typical feature of lipopolysaccharide (LPS)/D-galactosamine (D-Gal)-induced fulminant liver injury in mice, a well-established animal model for the investigation of dysregulated apoptosis of hepatocytes [19, 20]. In the present study, the expression of IRG1 and the production of itaconate were determined in mice with LPS/D-Gal-induced fulminant liver injury. And then, the significance of IRG1/itaconate in apoptotic liver injury was investigated in IRG1 knockout mice, and the underlying mechanisms were investigated with a signal pathway antibody microarray. Finally, 4-octyl itaconate (4-OI), a cell-permeable derivate of itaconate [14, 21], was supplemented, and its potential pharmacological value in the intervention of apoptotic liver injury was investigated.

## Methods

### Reagents

Lipopolysaccharide (from *Escherichia coli*, 055:B5) and D-galactosamine were from Sigma (St. Louis, MO, USA). 4-octyl itaconate (4-OI) was purchased from Cayman Chemical (Ann Arbor, Michigan, USA). The alanine aminotransferase (ALT) and aspartate aminotransferase (AST) assay kits were the products of Nanjing Jiancheng Bioengineering Institute (Nanjing, China). The caspase-3, -8, -9 colorimetric assay kits and the total protein extraction kit were from the Beyotime Institute of Biotechnology (Jiangsu, China). The AMP-activated protein kinase (AMPK) activator A-769662, 8-hydroxy-2-deoxy Guanosine (8-OH-dG), glutathione assay kit and thiobarbituric acid reactive substances (TBARS) assay kits were purchased from Cayman Chemical (Ann Arbor, Michigan, USA). Mouse Direct PCR Kit (for genotyping) was purchased from Bimake (Houston, Texas, USA). The antibodies for immune responsive gene 1 (IRG1, #17805S), AMPK (#5831T), phosphorylated AMPK (#2535S), c-jun-N-terminal kinase (JNK, #9252S), phosphorylated JNK (#4668T), cleaved caspase-3 (#9664S), β-actin (#3700S), HRP-linked anti-mouse (#7076S) and anti-rabbit (#7074S) antibodies were purchased from Cell Signaling Technology (Danvers, MA, United States). The antibodies for nuclear factor erythroid-2 related factor 2 (Nrf2, #16396-1-AP), heme oxygenase 1 (HO-1, #81281-1-RR) and NAD(P)H quinone oxyreductase (NQO1, #11451-1-AP) were purchased from Proteintech (Wuhan, China). The BCA protein assay kit and the enhanced chemiluminescence (ECL) reagents were produced by Thermo Fisher Scientific (Rockford, IL, USA).

### Mice

Male C57BL/6 mice (6–8 weeks old with a body weight of 18–22 g) were purchased from the Experimental Animal Center of Chongqing Medical University. IRG1^+/−^ mice were obtained from Cyagen Biosciences Inc. (Guangzhou, China) and self-mated to generate IRG1^−/−^ homozygous mice (IRG1 KO) and wild-type control mice. Both Alb-Cre and Nrf2 floxed (Nrf2^fl/fl^) mice were bought from the Jackson Laboratory. Hepatocyte-specific Nrf2^−/−^ (Nrf2^LKO^) mice were produced by backcrossing Nrf2^fl/fl^ and Alb-Cre mice. All mice were housed under specific pathogen-free conditions with controlled temperature and a 12 h light/dark cycle. Mice with sex, age (6–8 weeks old) and weight-matched (18–22 g) littermates were randomly allocated into different groups in experiments with no animal exclusion criteria. The sample size of each experiment was chosen based on previous experimental observations and indicated in the figure legend. All procedures involving animal subjects were performed in accordance with the Animal Research: Reporting of in vivo Experiments (ARRIVE) guidelines under the approval of the Ethics Committee of Chongqing Medical University.

### Experimental protocol

Fulminant liver injury was induced in mice by intraperitoneal injection of LPS (10 μg/kg) combined with D-Gal (700 mg/kg). To determine the level of IRG1 in liver tissue, the LPS/D-Gal-challenged mice were sacrificed at 0 h, 2 h, 4 h and 6 h post LPS/D-Gal exposure, the liver samples and serum samples were collected for the determination of IRG1 level and itaconate content, respectively. To investigate the cellular distribution of IRG1, hepatocytes and non-parenchymal cells were separated from the liver samples collected 6 h post LPS/D-Gal exposure, and the level of IRG1 was determined.

To investigate the potential roles of IRG1 in LPS/D-Gal-induced fulminant liver injury, wild-type and IRG1 KO mice were exposed to LPS/D-Gal. The animals were sacrificed at 6 h after LPS/D-Gal injection, and the liver samples and serum samples were collected for further experiments.

To investigate the potential significance of itaconate in LPS/D-Gal-induced fulminant liver injury, vehicle or 4-octyl itaconate (4-OI, 200 mg/kg, dissolved in DMSO) was administered intraperitoneally in LPS/D-Gal-challenged IRG1 KO mice. The animals were sacrificed at 6 h after LPS/D-Gal injection, and the liver samples and serum samples were collected for further experiments.

To investigate whether AMPK is involved in the hepatoprotective effects of 4-OI, A-769662 (30 mg/kg, dissolved in normal saline, i.p), an AMPK activator [22], was co-administered with 4-OI in C57BL/6 mice with LPS/D-Gal-induced liver injury. The animals were sacrificed at 6 h after LPS/D-Gal injection, and the liver samples and serum samples were collected for further experiments.

To investigate the potential roles of Nrf2 in mediating the protective benefits of itaconate, vehicle or 4-OI was administered intraperitoneally in LPS/D-Gal-challenged Nrf2^fl/fl^ mice or Nrf2^LKO^ mice. The animals were sacrificed at 6 h after LPS/D-Gal injection, and the liver samples and serum samples were collected for further experiments.

To investigate the therapeutic potential of 4-OI, vehicle or 4-OI was administered intraperitoneally 1.5 h post LPS/D-Gal exposure in C57BL/6 mice. The first set of animals was sacrificed at 6 h after LPS/D-Gal injection, and the liver samples and serum samples were collected for further experiments. Another set of animals was allocated, the survival of the experimental animals was recorded every 6 h for 7 days, and the survival rate of the mice was analyzed by the Kaplan–Meier curve.

### Gene Expression Omnibus (GEO) datasets analysis

To investigate the potential association between IRG1 and LPS/D-Gal-induced fulminant liver injury, a dataset GSE190823, containing the gene expression profiling data of liver samples from three normal mice and three mice with LPS/D-Gal-induced fulminant liver injury was downloaded from Gene Expression Omnibus (GEO, http://www.ncbi.nlm.nih.gov/geo/). Differentially expressed genes (DEGs) were screened with *P*-value < 0.05 and |log2FC| > 1 by using Sangerbox 3.0 (http://vip.sangerbox.com/).

### Separation of hepatocytes and nonparenchymal cells

Primary hepatocyte isolation and separation were performed by the two-step in-situ-circulating perfusion method, as reported previously [23]. Briefly, the mice were anesthetized, and the abdominal cavity was opened to expose the liver portal vein. The liver tissue was perfused with 0.05% collagenase (type VI; Sigma) through the portal vein for 20 min at 37 °C. Then, the liver tissue was excised, cut into small pieces, and pressed through a 70 µm nylon cell strainer. Finally, hepatocytes were separated from nonparenchymal cells by differential centrifugation.

### Determination of itaconate

The level of itaconate in the liver tissue was analyzed using an LC-MS/MS system as described previously [24]. Briefly, the supernatant was collected, 50 mg of the liver sample was homogenized with 1 ml of ice-cold methanol/water (80%, v/v), and the mixture was centrifuged at 12,000 rpm at 4 °C for 10 min. Subsequently, the obtained supernatant was dried under vacuum and then reconstituted in 0.1 ml solution [methanol/water/formic acid (15%:85%:0.1%)], which was centrifuged at 12,000 rpm for 10 min at 4 °C again to precipitate particulate matter. The supernatant was used for LC-MS/MS analysis, and two internal standards, itaconate and itaconic Acid-^13^C_5_,d4, were used to monitor the extraction efficiency.

### Real-time PCR

The details of the real-time PCR analysis were performed according to the MIQE guidelines. Total RNA from liver samples was extracted with the SteadyPure Quick RNA Extraction Kit (Accurate Biology) according to the manufacturer’s instructions, and the samples were stored at −80°. The purity of total RNA (A_260_/A_280_) was determined with NanoPhotometer N50 (IMPLEN). Isolated RNA was reverse-transcribed to cDNA using Evo M-MLV RT Mix Kit with gDNA Clean in accordance with the manufacturer’s instructions (Accurate Biology). The reaction volume for real-time PCR contained 2 μl of cDNA, 0.4 μl of each primer, 10 μl of 2X SYBR Green *Pro Taq*HS Premix (Accurate Biology) and RNase-free water for a total reaction volume of 20 μl. Real-time PCR was performed with CFX Connect equipment (BIO-RAD) with the following programs: pre-denaturation at 95 °C for 30 s, denaturation at 95 °C for 5 s (40 cycles), annealing at 60 °C for 30 s, and extension at 65 °C for 5 s. Finally, 95 °C for 50 s was performed for melting curve acquisition. The primer pairs for IRG1 (forward: 5′-3′: GCA ACA TGA TGC TCA AGT CTG and reverse: 5′-3′: TGC TCC TCC GAA TGA TAC CA) and glyceraldehyde-3-phosphate dehydrogenase (GAPDH) (forward: 5′-3′: TGT CCG TCG TGG ATC TGA C and reverse: 5′-3′: CCT GCT TCA CCA CCT TCT TG) were synthesized by Sangon Biotech (Shanghai, China). All samples were assayed in duplicate, the relative mRNA expression was normalized to GAPDH, and the results were assessed according to the 2^−^^∆∆Ct^ method.

### Western blot

The mouse livers were homogenized on ice in a lysis buffer (containing RIPA, EDTA, protease inhibitors and phosphatase inhibitors) to prepare tissue lysates. The lysates were centrifuged at 12,000 rpm for 10 min at 4 °C to collect the supernatant. The concentration of total protein was quantified by BCA assay. Samples were separated by electrophoresis on 7.5–12.5% SDS-PAGE and transferred to nitrocellulose membranes. The membranes were blocked for 2 h at room temperature with 5% low-fat milk in TBS with 0.05% Tween-20 (TBST) and then incubated with indicated primary antibodies at 4°C overnight. After incubation for 2 h at room temperature with the secondary antibody, the membranes were washed three times in TBST and exposed to the ChemiDoc Touch Imaging System (Bio-Rad). Relative quantification of band intensities was carried out using ImageJ software (US National Institutes of Health).

### Determination of transaminase

Serum samples were collected and analyzed for alanine aminotransferase (ALT) and aspartate aminotransferase (AST) levels with ALT and AST assay kits according to the manufacturer’s instructions (Nanjing Jiancheng).

### H&E and TUNEL staining

Liver tissues were fixed in a 4% paraformaldehyde solution. Liver sections (4 μm) were stained with hematoxylin and eosin (H&E). TUNEL staining was performed according to the manufacturer’s instructions (Roche). The TUNEL-positive apoptotic hepatocytes were counted at 400 magnification.

### Determination of the activity of caspases

The activities of caspase 3, caspase 8 and caspase 9 in liver tissue were measured using the caspase 3 colorimetric assay kit, caspase 8 colorimetric assay kit and caspase 9 colorimetric assay kit following the manufacturer’s instructions (Beyotime). Results were normalized by the total protein concentration of the supernatant.

### Determination of oxidative stress

The degree of lipid peroxidation in liver tissue was determined by Thiobarbituric Acid Reactive Substances (TBARS) assay kit from Cayman Chemical according to the protocol provided by the supplier. The protein concentration of the supernatant was determined by the BCA Protein Assay kit and used to normalize the TBARS content. In addition, the ratio of oxidized glutathione to glutathione (GSSG/GSH) was determined by Glutathione Assay Kit from Cayman Chemical according to the manufacturer’s instructions. For measures of DNA damage, the level of 8-hydroxy-2-deoxy Guanosine (8-OH-dG) in serum was performed by using DNA/RNA Oxidative Damage ELISA Kit from Cayman Chemical, according to the manufacturer’s protocol. The intensity measured was proportional to the amount of 8-OH-dG.

### Phospho-antibody microarray analysis

The signal pathway antibody microarray (CSP100 plus, Full Moon BioSystems) was used to analyze the potential signal pathway underlying the exacerbated liver injury in IRG1 KO mice. The antibody microarray consists of 304 antibodies, including 157 antibodies for phosphorylated proteins and 147 antibodies for unphosphorylated proteins involved in 16 signal pathways. Enrichment analysis of the data was performed using Sangerbox (http://sangerbox.com/).

### Statistical analysis

All data are expressed as mean and standard deviation (mean ± SD). Statistical analyses were performed with GraphPad Prism (Version: 8.0.1). The *t*-test was used to determine the difference between the means of the two groups. For multiple comparisons among means, the data were analyzed using one-way ANOVA with Tukey’s post hoc test. The survival analysis was performed using a Kaplan–Meier curve and log-rank test. The *P*-value < 0.05 was considered to be statistically significant.

## Results

### LPS/D-Gal-induced upregulation of IRG1 in liver

**Fig. 1:**
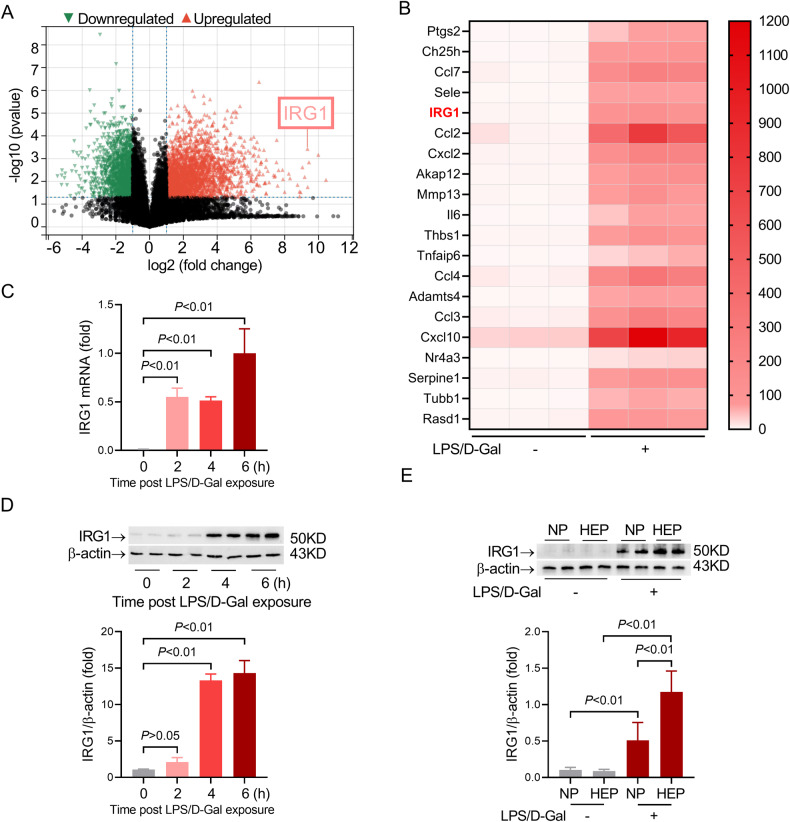
LPS/D-Gal-induced upregulation of hepatic IRG1. **A**, **B** The expression profiling data from the NCBI GEO database (GSE190823) was analyzed. **A** Volcano plots showing the DEGs (*P*-value < 0.05 and |log2 fold change (FC)| > 1) between LPS/D-Gal-insulted liver and normal control liver. **B** The most highly upregulated 20 genes were shown. **C**–**E** Mice with LPS/D-Gal-induced fulminant liver injury were sacrificed at the indicated time points. The levels of **C** IRG1 mRNA and **D** IRG1 protein were determined (*n* = 4). **E** Non-parenchymal cells (NP) and hepatocytes (HEP) were separated from the liver samples collected 6 h post LPS/D-Gal exposure, and the levels of IRG1 protein in NP and HEP were determined (*n* = 4). All data were expressed as mean ± SD.

To investigate the potential association between IRG1 and LPS/D-Gal-induced liver injury, the expression profiling data from the NCBI GEO database (GSE190823) was analyzed. There is a total of 4042 differentially expressed genes (DEGs) in the liver after LPS/D-Gal exposure, including 1719 downregulated DEGs and 2323 upregulated DEGs (Fig. 1A), and IRG1 is one of the most highly upregulated genes (Fig. 1B). Consistently, the present study found that LPS/D-Gal exposure significantly upregulated the mRNA and protein levels of IRG1 in the liver (Fig. 1C, D). To further investigate the cellular distribution of IRG1, hepatocytes and non-parenchymal cells were separated, and the immunoblot analysis found that LPS/D-Gal induced upregulation of IRG1 in both hepatocytes and non-parenchymal cells, but the level of IRG1 in hepatocytes was much higher than that in non-parenchymal cells (Fig. 1E).

### Deletion of IRG1 aggravates LPS/D-Gal-induced fulminant liver injury

**Fig. 2:**
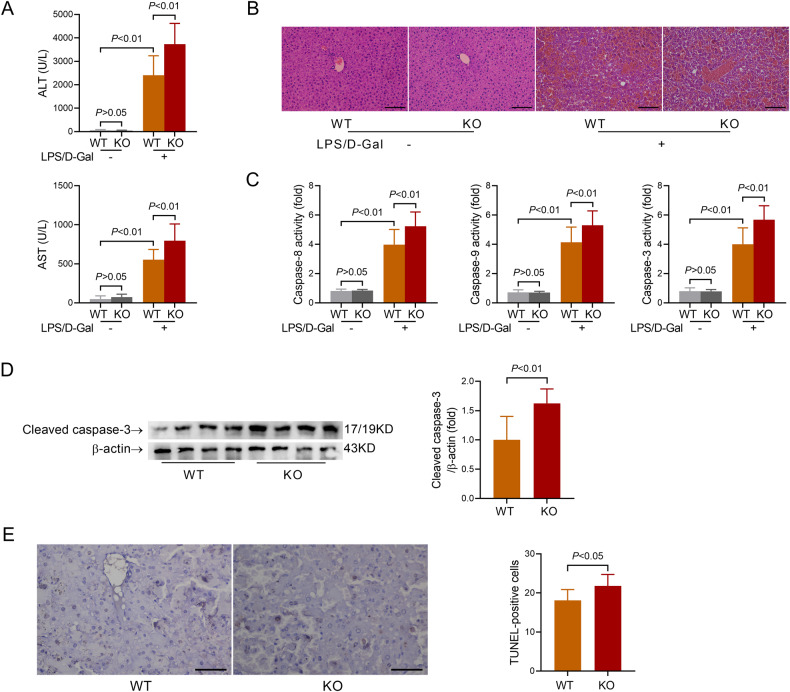
IRG1 deficiency aggravated LPS/D-Gal-induced fulminant liver injury. WT or IRG1 KO mice with fulminant liver injury were sacrificed 6 h post LPS/D-Gal exposure. **A** The serum levels of ALT and AST were determined (*n* = 8). **B** The liver sections were stained with hematoxylin & eosin for histological examination (scale bar: 100 μm). **C** The hepatic activities of caspase-8, caspase-9 and caspase-3 were determined (*n* = 8). **D** The hepatic level of cleaved caspase-3 was detected (*n* = 4). **E** The apoptotic hepatocytes were evaluated by TUNEL staining (scale bar: 50 μm), and the TUNEL-positive cells were counted. All data were expressed as mean ± SD.

To investigate the potential significance of upregulated IRG1, IRG1 knockout (KO) mice and wild-type (WT) mice were produced (Supplementary Fig. 1A, B). In IRG1 KO mice challenged with LPS/D-Gal, the induction of IRG1 was abolished (Supplementary Fig. 1C, D). Deletion of IRG1 did not result in the elevation of aminotransferases in serum in vehicle-treated mice (Fig. 2A), but the elevation of ALT/AST was higher in IRG1 KO mice than that in WT mice after LPS/D-Gal exposure (Fig. 2A). In histological examination, deletion of IRG1 did not result in any obvious histological abnormalities in vehicle-treated mice, the morphology of liver cells in IRG1 KO mice was regular, and the hepatocyte was arranged neatly, without obvious steatosis, degeneration or inflammatory cell infiltration (Fig. 2B). However, LPS/D-Gal induced more severe histological abnormalities in the liver of IRG1 KO mice (Fig. 2B).

The anti-inflammatory activities of IRG1 have been well-documented [25]. The present study also found that the deletion of IRG1 further increased the serum levels of TNF-α and IL-6 in LPS/D-Gal-insulted mice (Supplementary Fig. 2A, B). Massive hepatocyte apoptosis has been suggested as a typical feature of LPS/D-Gal-induced fulminant liver injury [19]. The present study found that deletion of IRG1 did not result in any significant alteration of caspase cascade in vehicle-treated mice (Fig. 2C–E). However, the activation of the caspase cascade (Fig. 2C), the cleavage of caspase-3 (Fig. 2D), and the abundance of TUNEL-positive cells (Fig. 2E) were all enhanced in IRG1 KO mice with LPS/D-Gal exposure. These results indicate that IRG1 might function as a protective enzyme in LPS/D-Gal-induced fulminant liver injury.

### IRG1 deficiency promotes hepatocyte apoptosis via enhancing AMPK/JNK activation

**Fig. 3:**
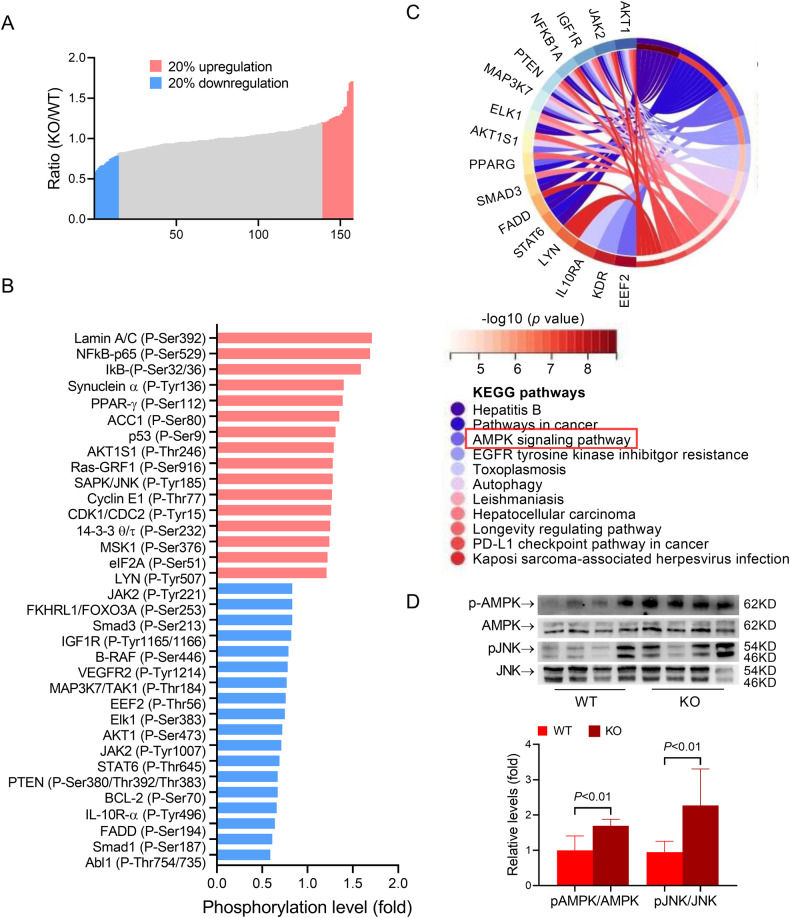
IRG1 deficiency enhanced phosphorylation of AMPK/JNK. WT or IRG1 KO mice with fulminant liver injury were sacrificed 6 h post LPS/D-Gal exposure. **A**–**C** The signal pathway antibody microarray (CSP100 plus) was used to identify the potential signaling pathways. **A**, **B** The proteins whose phosphorylation levels were upregulated or downregulated by more than 20% were labeled red or blue, respectively. **C** Chord plots show the enrichment of the differentially phosphorylated proteins by Kyoto Encyclopedia of Genes and Genomes (KEGG) analyses. **D** The hepatic levels of phosphorylated AMPK (pAMPK), total AMPK (AMPK), phosphorylated JNK (pJNK), and total JNK (JNK) were determined (*n* = 4). All data were expressed as mean ± SD.

To investigate the potential mechanisms underlying the aggravated liver injury in IRG1 KO mice, a signal pathway antibody microarray (CSP100 plus) was used to identify the potential signaling pathways. The antibody microarray analysis found that the levels of 19 phosphorylated proteins were upregulated, and the levels of 14 phosphorylated proteins were downregulated in IRG1 KO mice, as compared to those in WT mice (Fig. 3A, B). Interestingly, enrichment analysis indicated that AMP-activated protein kinase (AMPK) signaling pathway might be involved in the aggravated liver injury in IRG1 KO mice (Fig. 3C). Immunoblot analysis indicated that LPS/D-Gal-induced phosphorylation of AMPK was significantly enhanced in IRG1 KO mice (Fig. 3D). Previous studies found that c-jun-N-terminal kinase (JNK) was a major downstream target responsible for the pro-apoptotic property of AMPK in LPS/D-Gal-induced fulminant liver injury [22]. In the present study, IRG1 deletion also resulted in enhanced phosphorylation of JNK in LPS/D-Gal-exposed mice (Fig. 3D). These data suggested that the activation of AMPK/JNK might be negatively regulated by IRG1.

To investigate the significance of the AMPK/JNK pathway in the exacerbated liver injury in IRG1 KO mice, a widely used AMPK inhibitor, compound C [26], or SP600125, a widely used JNK inhibitor [27], was administered in IRG1 KO mice exposed to LPS/D-Gal. The result indicated that treatment with compound C or SP600125 resulted in decreased phosphorylation levels of AMPK and/or JNK in LPS/D-Gal-challenged IRG1 KO mice (Supplementary Figs. 3A and 4A). In addition, the enhanced cleavage of caspase-3 and activation of caspase cascade, the increased abundance of TUNEL-positive cells, the exacerbated elevation of ALT and AST, and the aggravated induction of histological abnormalities in LPS/D-Gal-challenged IRG1 KO mice were reversed by compound C or SP600125 (Supplementary Figs. 3A–E and 4A–E). Thus, the enhanced activation of the AMPK/JNK pathway might be crucial molecular events that are pathologically involved in the aggravation of liver injury in IRG1 KO mice.

### IRG1 deficiency enhances AMPK/JNK activation via the reduction of itaconate production

**Fig. 4:**
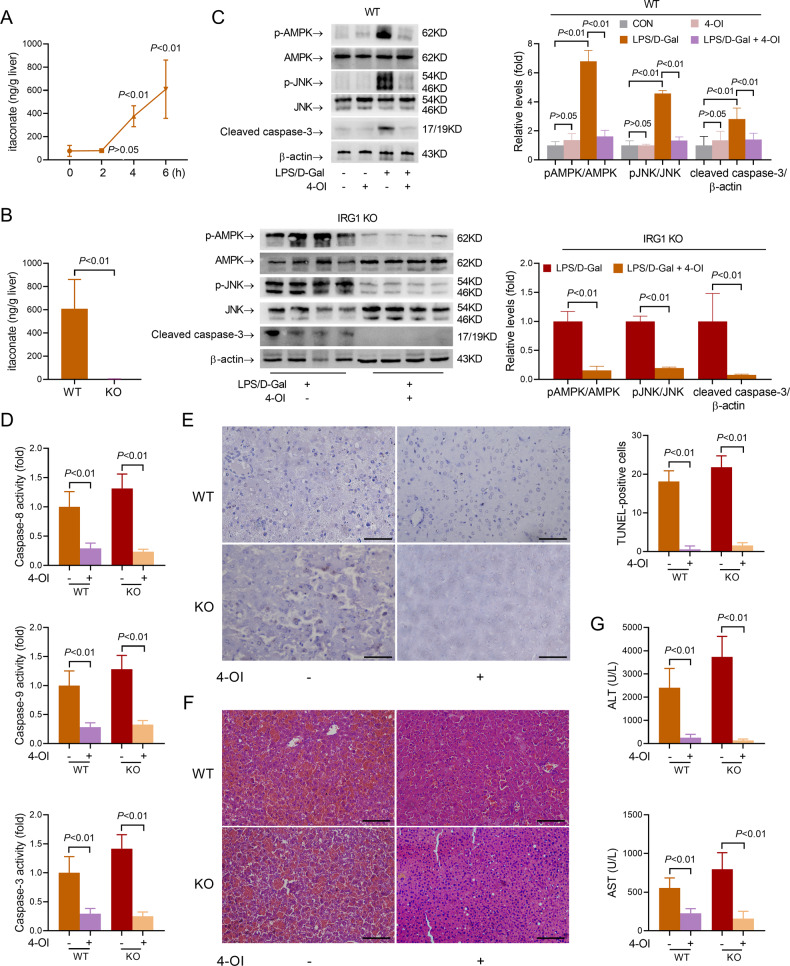
Reduction of itaconate resulted in aggravated liver injury in IRG1 KO mice. **A** Mice with LPS/D-Gal-induced fulminant liver injury were sacrificed at the indicated time points. The levels of itaconate were determined (*n* = 8). **B** WT and IRG1 KO mice with fulminant liver injury were sacrificed 6 h post LPS/D-Gal exposure. The levels of itaconate (*n* = 8) were determined. **C**–**G** WT or IRG1 KO mice with fulminant liver injury were supplemented with 4-octyl itaconate (4-OI) and sacrificed 6 h post LPS/D-Gal exposure. **C** The hepatic levels of phosphorylated AMPK (pAMPK), total AMPK (AMPK), phosphorylated JNK (pJNK), total JNK (JNK) and cleaved caspase-3 were determined (*n* = 4). **D** The hepatic activities of caspase-8, caspase-9 and caspase-3 were determined (*n* = 8). **E** The apoptotic hepatocytes were evaluated by TUNEL staining (scale bar: 50 μm), and the TUNEL-positive cells were counted. **F** The liver sections were stained with hematoxylin & eosin for histological examination (scale bar: 100 μm). **G** The serum level of ALT and AST were determined (*n* = 8). All data were expressed as mean ± SD.

We then questioned whether deletion of IRG1 enhances AMPK/JNK activation and exacerbates liver injury via reduction of itaconate production. Consistent with the upregulation of IRG1, the production of itaconate increased after LPS/D-Gal exposure (Fig. 4A). In IRG1 KO mice, the level of itaconate could hardly be detected (Fig. 4B). Supplementation with 4-OI, a cellular permeable derivate of itaconate [14, 21], resulted in suppressed phosphorylation of AMPK and JNK in LPS/D-Gal-challenged C57BL/6 mice (Fig. 4C). Consistently, supplementation with 4-OI also reversed the enhanced AMPK/JNK phosphorylation in LPS/D-Gal-challenged IRG1 KO mice (Fig. 4C), suggesting that the activation of AMPK/JNK pathway in liver injury would be negatively modulated by itaconate. Treatment with 4-OI alone has little effect on caspase-3 activity or ALT/AST level (Supplementary Fig. 5A, B), while supplementation with 4-OI in both WT and IRG1 KO mice suppressed LPS/D-Gal-induced cleavage of caspase-3, activation of caspase cascade, presence of TUNEL-positive apoptotic cells, induction of histological abnormalities and elevation of ALT/AST (Fig. 4C–G). These results suggest that reduced production of itaconate might be involved in the aggravated liver injury in IRG1 KO mice.

**Fig. 5:**
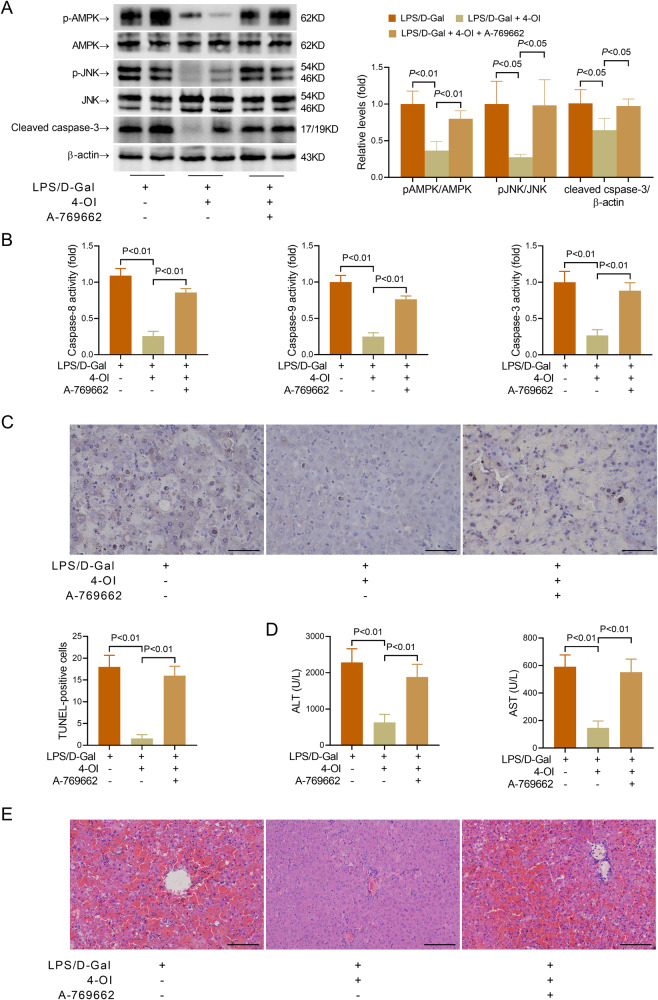
AMPK activator reversed the suppressive effects of 4-octyl itaconate on liver injury. Mice with fulminant liver injury were supplemented with 4-octyl itaconate (4-OI) and AMPK activator A-769662 and sacrificed 6 h post LPS/D-Gal exposure. **A** The hepatic levels of phosphorylated AMPK (pAMPK), total AMPK (AMPK), phosphorylated JNK (pJNK), total JNK (JNK), and cleaved caspase-3 were detected (*n* = 4). **B** The hepatic activities of caspase-8, caspase-9 and caspase-3 were determined (*n* = 8). **C** The apoptotic hepatocytes were evaluated by TUNEL staining (scale bar: 50 μm), and the TUNEL-positive cells were counted. **D** The serum level of ALT and AST were determined (*n* = 8). **E** The liver sections were stained with hematoxylin & eosin for histological examination (scale bar: 100 μm). All data were expressed as mean ± SD.

To further investigate the pathological association between AMPK and itaconate, A-769662, an AMPK activator [22], was co-administered with 4-OI in LPS/D-Gal-challenged C57BL/6 mice. As expected, co-administration of A-769662 reversed the suppressive effects of 4-OI on AMPK/JNK phosphorylation (Fig. 5A). In addition, the alleviated hepatic apoptosis and the histological abnormalities in 4-OI-treated mice were abolished by A-769662 (Fig. 5A–E). These results suggest that suppression of the AMPK/JNK pathway might be responsible for the anti-apoptotic benefits of itaconate in mice with LPS/D-Gal-induced liver injury.

### IRG1/Itaconate modulates liver injury in an Nrf2-dependent manner

**Fig. 6:**
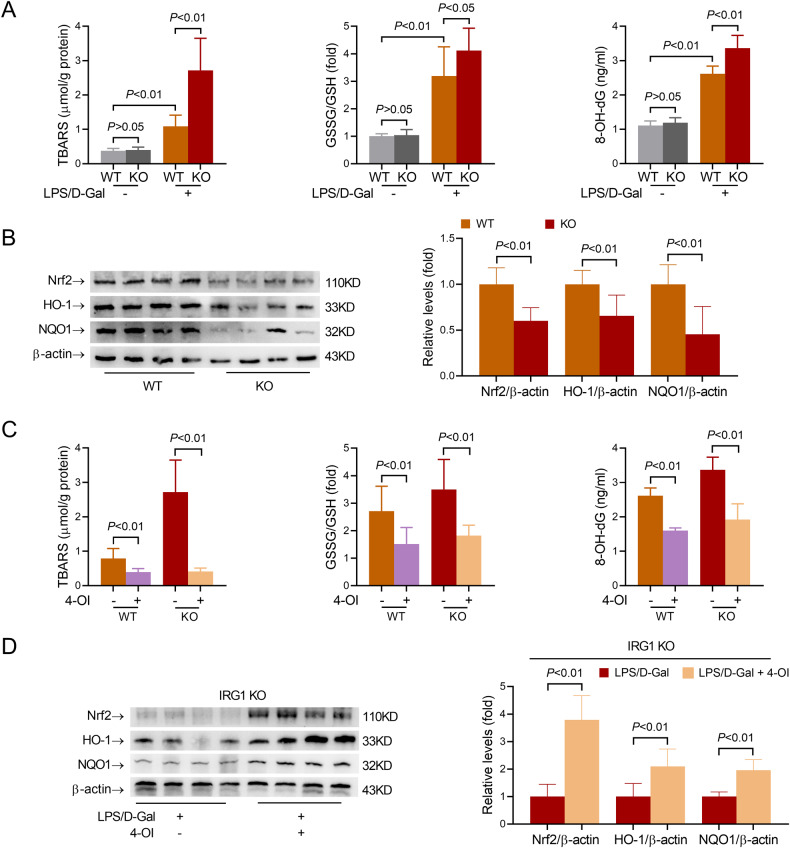
Nrf2 pathway and oxidative stress were associated with IRG1/itaconate in fulminant liver injury. **A**, **B** WT or IRG1 KO mice with fulminant liver injury were sacrificed 6 h post LPS/D-Gal exposure. **A** The hepatic contents of TBARS, the GSSG/GSH ratio in the liver, and the serum level of 8-OH-dG were determined (*n* = 8). **B** The protein levels of Nrf2, HO-1, and NQO1 in liver samples were determined (*n* = 4). **C**, **D** WT or IRG1 KO mice with fulminant liver injury were supplemented with 4-octyl itaconate (4-OI) and sacrificed 6 h post LPS/D-Gal exposure. **C** The hepatic contents of TBARS, GSSG/GSH ratio in the liver, and the serum level of 8-OH-dG were determined (*n* = 8). **D** The protein levels of Nrf2, HO-1, and NQO1 in liver samples were determined (*n* = 4). All data were expressed as mean ± SD.

Previous studies have found that the anti-oxidative nuclear factor erythroid-2 related factor 2 (Nrf2) is a main target responsible for the bioactivities of itaconate [21]. In the present study, the aggravated liver injury in IRG1 KO mice was associated with a higher level of TBARS, a widely used marker of oxidative stress [28], a higher ratio of GSSG/GSH, and a higher level of 8-OH-dG, a biomarker of oxidative DNA damage [29] (Fig. 6A). Exposure to LPS/D-Gal also resulted in decreased levels of Nrf2 and its downstream targets, including HO-1 and NQO1 in IRG1 KO mice (Fig. 6B). Treatment with 4-OI alone has little effect on hepatic TBARS, GSSG/GSH and 8-OH-dG (Supplementary Fig. 5C), while supplementation with 4-OI suppressed LPS/D-Gal-induced elevation of TBARS, GSSG/GSH and 8-OH-dG in both WT and IRG1 KO mice (Fig. 6C). In addition, supplementation with 4-OI in IRG1 KO mice significantly reversed LPS/D-Gal-induced reduction of Nrf2, HO-1, and NQO1 in liver (Fig. 6D), suggesting that the suppressed Nrf2 signaling and enhanced oxidative stress might be associated with the deficiency of itaconate in IRG1 KO mice.

**Fig. 7:**
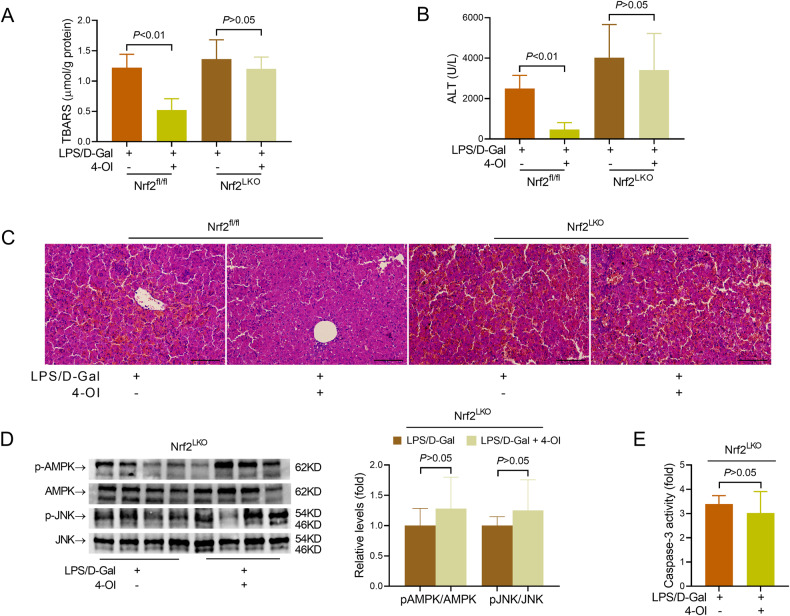
The anti-apoptotic benefits of 4-octyl itaconate depended on hepatic Nrf2. **A**–**E** Nrf2^fl/fl^ or Nrf2^LKO^ mice with fulminant liver injury were supplemented with 4-octyl itaconate (4-OI) and sacrificed 6 h post LPS/D-Gal exposure. **A** The hepatic content of TBARS was determined. **B** The serum level of ALT was determined. **C** The liver sections were stained with hematoxylin & eosin for histological examination (scale bar: 100 μm). **D** The hepatic levels of phosphorylated AMPK (pAMPK), total AMPK (AMPK), phosphorylated JNK (pJNK) and total JNK (JNK) were determined (*n* = 4). **E** The hepatic activity of caspase-3 was determined (*n* = 8). All data were expressed as mean ± SD.

Because IRG1 is mainly upregulated in hepatocytes (as shown in Fig. 1E), the hepatocyte-specific Nrf2 knockout mice (Nrf2^LKO^) were produced to investigate the significance of hepatic Nrf2 in the anti-apoptotic benefits of itaconate (Supplementary Fig. 6A–C). The ALT and AST levels in serum and the histological structure of the liver in Nrf2^LKO^ mice were comparable to those in Nrf2^fl/fl^ mice (Supplementary Fig. 6D, E). Interestingly, treatment with 4-OI resulted in a decreased level of TBARS and suppressed elevation of ALT in Nrf2^fl/fl^ mice (Fig. 7A, B), but 4-OI failed to attenuate oxidative stress, suppress ALT elevation, or alleviate histological abnormalities in Nrf2^LKO^ mice (Fig. 7A–C). In addition, the suppressive effects of 4-OI on AMPK/JNK signaling and caspase-3 activation were absent in Nrf2^LKO^ mice (Fig. 7D, E). Therefore, the anti-apoptotic effects of 4-OI in the present study might depend on the activation of Nrf2 in hepatocytes.

To further investigate the significance of oxidative stress in the enhanced activation of AMPK and exacerbated liver injury in IRG1 KO mice, N-acetylcysteine (NAC), a representative antioxidant [30], was administered in IRG1 KO mice with LPS/D-Gal exposure. As expected, supplementation with NAC significantly decreased the level of TBARS, GSSG/GSH and 8-OH-dG in LPS/D-Gal-challenged IRG1 KO mice (Supplementary Fig. 7A). Supplementation with NAC also resulted in compromised AMPK/JNK signaling (Supplementary Fig. 7B). In addition, the cleavage of caspase-3 and the activation of caspase cascade, the increase of TUNEL-positive cells, the elevation of ALT and AST, and the induction of histological abnormalities were suppressed by NAC (Supplementary Fig. 7B–F). Thus, the enhanced oxidative stress might be crucial for the enhanced AMPK/JNK activation and the aggravated apoptotic liver injury in IRG1 KO mice.

### Post-insult supplementation with 4-OI alleviates LPS/D-Gal-induced fulminant liver injury

**Fig. 8:**
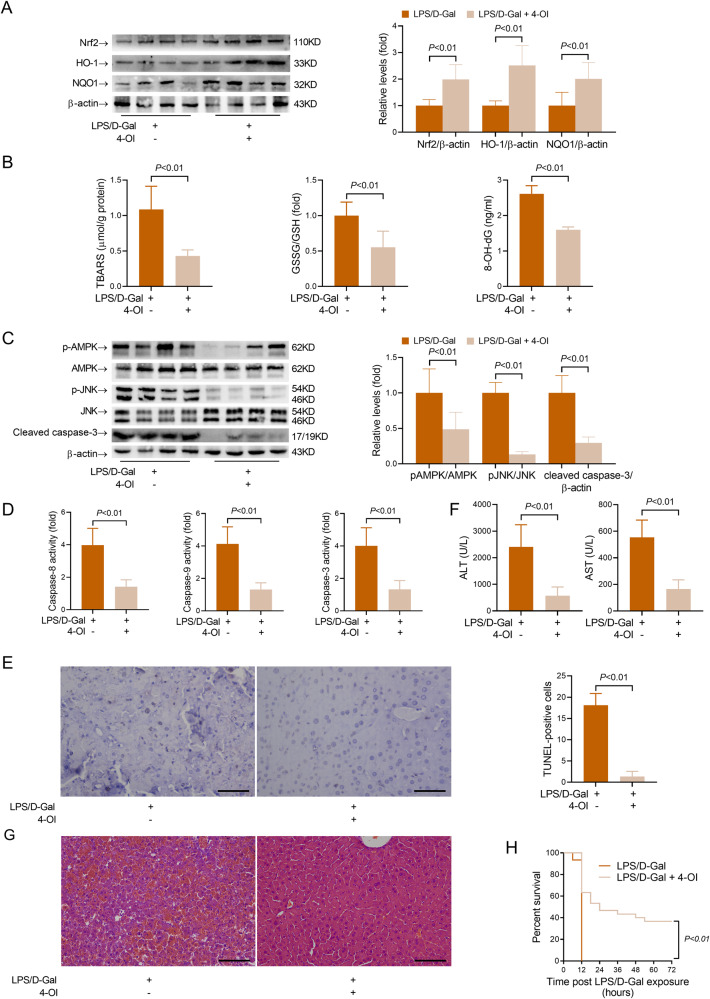
Post-treatment with 4-octyl itaconate resulted in beneficial outcomes in mice with fulminant liver injury. 4-octyl itaconate (4-OI) was administered in mice with fulminant liver injury 1.5 h post LPS/D-Gal exposure, and the animals were sacrificed 6 h post LPS/D-Gal exposure. **A** The protein levels of Nrf2, HO-1 and NQO1 in liver samples were determined (*n* = 4). **B** The hepatic contents of TBARS, the ratio of GSSG/GSH in the liver and the serum level of 8-OH-dG were determined (*n* = 8). **C** The hepatic levels of phosphorylated AMPK (pAMPK), total AMPK (AMPK), phosphorylated JNK (pJNK), total JNK (JNK) and cleaved caspase-3 were determined (*n* = 4). **D** The hepatic activities of caspase-8, caspase-9 and caspase-3 were determined (*n* = 8). **E** The apoptotic hepatocytes were evaluated by TUNEL staining (scale bar: 50 μm), and the TUNEL-positive cells were counted. **F** The serum levels of ALT and AST were determined (*n* = 8). **G** The liver sections were stained with hematoxylin & eosin for histological examination (scale bar: 100 μm). **H** The survival rate of the experimental animals was monitored and showed in the Kaplan–Meier curves (*n* = 20). All data were expressed as mean ± SD.

To investigate the pharmacological potential of the itaconate-AMPK pathway, 4-OI was administered post LPS/D-Gal exposure. The results indicated that post-insult supplementation with 4-OI upregulated Nrf2, HO-1 and NQO1 but decreased the level of TBARS, GSSG/GSH and 8-OH-dG (Fig. 8A, B). Post-insult supplementation with 4-OI also suppressed the phosphorylation of AMPK and JNK (Fig. 8C). Most importantly, post-insult supplementation with 4-OI suppressed the cleavage of caspase-3 and the activation of caspase cascade, the induction of TUNEL-positive cells, the elevation of ALT and AST, the development of histological abnormalities and improved the survival rate of LPS/D-Gal-challenged mice (Fig. 8C–H). Thus, 4-OI might have potential pharmacological value for the therapy of fulminant liver injury.

## Discussion

An increasing number of evidence suggests that IRG1-catalyzed generation of itaconate functions as a protective metabolic event under pathological circumstances [25]. Previous studies have found that IRG1/itaconate protected experimental animals from allergic asthma, pulmonary fibrosis, and ischemia-reperfusion injury [23, 31, 32]. In the present study, we found that the expression of IRG1 and its product itaconate were significantly increased in mice with LPS/D-Gal-induced fulminant liver injury. Deletion of IRG1 resulted in exacerbated hepatocytes apoptosis and aggravated liver injury in mice, but supplementation of 4-OI attenuated liver injury. Therefore, the upregulation of IRG1/itaconate might be a novel metabolic mechanism that protects hepatocytes from apoptosis in fulminant liver injury.

The electrophilic α, β-unsaturated carboxylic acid group within itaconate directly modifies the cysteine residues on several proteins involved in signal transduction and metabolic reactions [13, 14]. Kelch-like ECH-associated protein 1 (KEAP1), the master regulator of Nrf2, is one of the major targets responsible for the protective benefits of itaconate [21, 33]. Modification of KEAP1 by itaconate prevented Nrf2 from degradation through the ubiquitin-proteasome system, which led to the activation of the Nrf2 antioxidant pathway [21]. Previous studies have found that oxidative stress plays an essential role in the induction of hepatocyte apoptosis in LPS/D-Gal-challenged mice [34, 35]. Nrf2 is a major regulator of redox balance, which protects the hepatocytes under various pathological circumstances [36–38]. In the present study, the deletion of IRG1 resulted in suppressed activation of the Nrf2 pathway and enhanced oxidative stress, which would be reversed by the supplementation of 4-OI. Thus, the Nrf2 pathway might be associated with the protective effects of IRG1/itaconate in fulminant liver injury.

IRG1 has been identified as one of the most highly upregulated genes under pro-inflammatory conditions in macrophages [39]. In addition to macrophage, recent studies have found that IRG1 was also expressed in neurons, chondrocytes, hepatocytes as well as tumor cells [23, 40–42]. The present study found that the upregulation of IRG1 is much higher in hepatocytes than that in non-parenchymal cells in mice with LPS/D-Gal-induced fulminant liver injury, suggesting that hepatocytes might be the major cellular targets responsible for the protective benefits of IRG1/itaconate in fulminant liver injury. Interestingly, the present study found that supplementation with 4-OI failed to provide anti-apoptotic benefits in mice with liver-specific deletion of Nrf2. Therefore, activation of Nrf2 anti-oxidative pathway in hepatocytes might be a pivotal mechanism underlying the anti-apoptotic activities of IRG1/itaconate in liver.

In addition to the direct induction of functional disturbance or structural damage in cells, the activation of redox-sensitive signal pathways significantly amplifies the detrimental effects of oxidative stress[43, 44]. The redox-sensitive AMPK has been identified as a late-stage pro-apoptotic factor in LPS/D-Gal-induced fulminant liver injury via phosphorylation of JNK [22, 45], which might have therapeutic value for fulminant liver injury. The present study found that the exacerbated liver injury in mice lacking IRG1 was associated with enhanced phosphorylation of AMPK and JNK, which was reversed by the supplementation of 4-OI. In addition, treatment with the AMPK inhibitor compound C suppressed the enhanced phosphorylation of AMPK/JNK and alleviated the aggravated hepatocyte apoptosis in IRG1 KO mice. In line with our findings, the AMPK/JNK pathway is involved in oxidative stress-induced apoptosis of myocardial cells, myeloma cells and pancreatic beta cells [46–48]. Therefore, the upregulation of IRG1/itaconate might function as a molecular brake that negatively regulates the late-stage pro-apoptotic AMPK/JNK signaling.

The oxidative stress might promote the activation of AMPK via direct oxidative modification of AMPK [49, 50]. A previous study has found that Nrf2 deficiency resulted in enhanced activation of AMPK in skeletal muscle [51]. In addition, the knockdown of Nrf2 by shRNA induced phosphorylation of AMPK in glioma cells [52]. In the present study, although supplementation with 4-OI reversed the enhanced activation of AMPK/JNK in IRG1 KO mice, it failed to modulate the phosphorylation of AMPK and JNK in mice lacking Nrf2. On the contrary, treatment with the antioxidant NAC, suppressed AMPK and JNK phosphorylation and alleviated apoptotic liver injury in IRG1 KO mice. Therefore, the suppressive effects of IRG1/itaconate on the AMPK/JNK pathway might depend on the activation of Nrf2-dependent antioxidant defense.

Because the upregulation of IRG1/itaconate might be a novel endogenous protective response that is involved in the control of the late-stage pro-apoptotic AMPK/JNK signaling, we then questioned whether 4-OI might have pharmacological value for the intervention of fulminant liver injury. Interestingly, the present study found that post-insult treatment with 4-OI significantly enhanced Nrf2 signaling but inhibited the activation of AMPK/JNK. Post-treatment with 4-OI also blunted the activation of the caspase cascade, reduced the count of TUNEL-positive cells, suppressed the elevation of ALT, alleviated the histological abnormalities, and improved the survival rate of the experimental animals. Therefore, 4-OI might have promising value for the pharmacological therapy of fulminant liver injury.

In addition to KEAP1, itaconate also directly alkylates the glycolytic enzyme glyceraldehyde-3-phosphate dehydrogenase (GAPDH) and decreases its enzyme activity, which resulted in inhibition of glycolysis [14]. Glycolysis is a crucial metabolic basis for various physiological activities and pathological responses, and previous studies have found that pharmacological inhibition of glycolysis resulted in beneficial outcomes in acute liver injury [53]. Thus, whether GAPDH is involved in the hepatoprotective of itaconate might be worthy of further investigation. Several recent studies have also found that Janus kinase 1(JAK1), Nod-like receptor 3 (NLRP3), and transcription factor EB (TFEB) were directly alkylated by itaconate, which is profoundly involved in the bioactivities of itaconate [54–56], whether these targets are also involved in the hepatoprotective of itaconate remain to be investigated.

Interestingly, several studies have found that itaconate is secreted by activated macrophages [57, 58]. Although the molecular mechanisms through which itaconate is secreted have not been revealed, a recent study has identified that 2-oxoglutarate receptor 1 (OXGR1), a G protein-coupled receptor also known as GPR99, functions as a receptor for itaconate that induces calcium mobilization and mucus secretion in the respiratory epithelium [59]. Thus, in addition to intracellular production and alkylation, itaconate is secreted and functions as an extracellular signal molecule to communicate with other cells, whether the itaconate/OXGR1 signal pathway is involved in the development of fulminant liver injury remains to be further investigated.

**Fig. 9:**
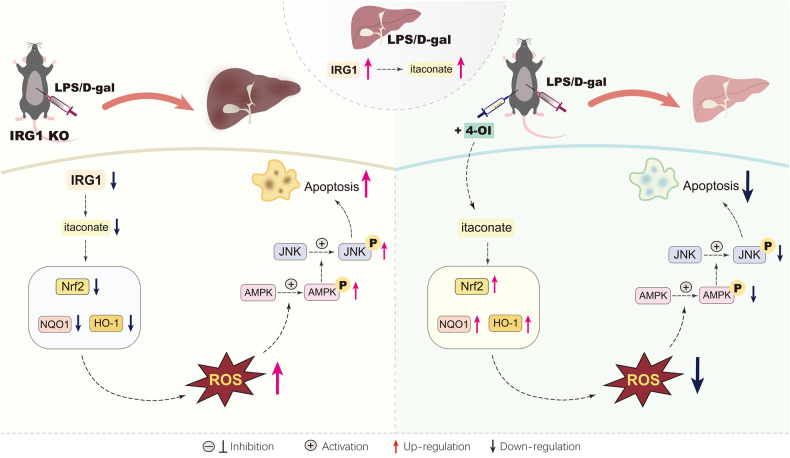
The schematic diagram of the mechanisms underlying the anti-apoptotic benefits of IRG1/itaconate in LPS/D-gal-induced fulminant liver injury. LPS/D-gal exposure induced the upregulation of IRG1 and itaconate. Genetic deletion of IRG1 resulted in compromised activation of Nrf2, decreased the expression of anti-oxidative enzymes, such as NQO1 and HO-1, and enhanced oxidative stress. Then, the redox-sensitive AMPK/JNK pathway was activated, which promoted the apoptosis of hepatocytes and aggravated liver injury. Supplementation with 4-OI, a cell-permeable derivate of itaconate, promoted the activation of the anti-oxidative Nrf2 pathway, suppressed oxidative stress, inhibited the activation of the pro-apoptotic AMPK/JNK pathway, attenuated hepatic apoptosis, and alleviated liver injury.

Taken together, the present study found that the upregulation of IRG1/itaconate in mice with LPS/D-Gal-induced fulminant liver injury might be an endogenous protective response, which might result from the activation of Nrf2 signaling and the subsequent suppression of oxidation-sensitive activation of AMPK/JNK (Fig. 9). Most importantly, the present study found that post-insult supplementation with the itaconate derivate 4-OI also resulted in beneficial outcomes (Fig. 9). Therefore, IRG1/itaconate might function as a metabolic brake in fulminant liver injury, and itaconate derivates might have potential value for the pharmacological intervention of fulminant liver injury.

## Supplementary information

supplementary information

Supplementary figure 1

Supplementary figure 2

Supplementary figure 3

Supplementary figure 4

Supplementary figure 5

Supplementary figure 6

Supplementary figure 7

Reproducibility checklist

Original Data File

## Data Availability

The data generated in this study are available upon request from the corresponding author.

## References

[1] Schwabe RF, Luedde T (2018). Apoptosis and necroptosis in the liver: a matter of life and death. Nat Rev Gastroenterol Hepatol.

[2] Kumar V, Xin X, Ma J, Tan C, Osna N, Mahato RI (2021). Therapeutic targets, novel drugs, and delivery systems for diabetes associated NAFLD and liver fibrosis. Adv Drug Deliv Rev.

[3] Cao L, Quan XB, Zeng WJ, Yang XO, Wang MJ (2016). Mechanism of hepatocyte apoptosis. J Cell Death.

[4] Kesavardhana S, Malireddi RKS, Kanneganti TD (2020). Caspases in cell death, inflammation, and pyroptosis. Annu Rev Immunol.

[5] Wiley CD, Campisi J (2021). The metabolic roots of senescence: mechanisms and opportunities for intervention. Nat Metab.

[6] Green DR, Galluzzi L, Kroemer G (2014). Cell biology. Metabolic control of cell death. Science.

[7] Gaber T, Strehl C, Buttgereit F (2017). Metabolic regulation of inflammation. Nat Rev Rheumatol.

[8] Madden MZ, Rathmell JC (2021). The complex integration of T-cell metabolism and immunotherapy. Cancer Discov.

[9] Wang H, Yang Y, Liu J, Qian L (2021). Direct cell reprogramming: approaches, mechanisms and progress. Nat Rev Mol Cell Biol.

[10] Li F, Xu W, Zhao S (2013). Regulatory roles of metabolites in cell signaling networks. J Genet Genomics.

[11] Murphy MP, O’Neill LAJ (2018). Krebs cycle reimagined: the emerging roles of succinate and itaconate as signal transducers. Cell..

[12] Chen M, Sun H, Boot M, Shao L, Chang SJ, Wang W (2020). Itaconate is an effector of a Rab GTPase cell-autonomous host defense pathway against *Salmonella*. Science..

[13] Qin W, Zhang Y, Tang H, Liu D, Chen Y, Liu Y (2020). Chemoproteomic profiling of itaconation by bioorthogonal probes in inflammatory macrophages. J Am Chem Soc.

[14] Liao ST, Han C, Xu DQ, Fu XW, Wang JS, Kong LY (2019). 4-Octyl itaconate inhibits aerobic glycolysis by targeting GAPDH to exert anti-inflammatory effects. Nat Commun.

[15] Chen LL, Morcelle C, Cheng ZL, Chen X, Xu Y, Gao Y (2022). Itaconate inhibits TET DNA dioxygenases to dampen inflammatory responses. Nat Cell Biol.

[16] Zheng Y, Chen Z, She C, Lin Y, Hong Y, Shi L (2020). Four-octyl itaconate activates Nrf2 cascade to protect osteoblasts from hydrogen peroxide-induced oxidative injury. Cell Death Dis.

[17] Liu H, Feng Y, Xu M, Yang J, Wang Z, Di G (2018). Four-octyl itaconate activates Keap1-Nrf2 signaling to protect neuronal cells from hydrogen peroxide. Cell Commun Signal.

[18] Tang C, Tan S, Zhang Y, Dong L, Xu Y (2019). Activation of Keap1-Nrf2 signaling by 4-octyl itaconate protects human umbilical vein endothelial cells from high glucose. Biochem Biophys Res Commun.

[19] Wu J, Zhao Y, Park YK, Lee JY, Gao L, Zhao J (2018). Loss of PDK4 switches the hepatic NF-kappaB/TNF pathway from pro-survival to pro-apoptosis. Hepatology..

[20] Amir M, Zhao E, Fontana L, Rosenberg H, Tanaka K, Gao G (2013). Inhibition of hepatocyte autophagy increases tumor necrosis factor-dependent liver injury by promoting caspase-8 activation. Cell Death Differ.

[21] Mills EL, Ryan DG, Prag HA, Dikovskaya D, Menon D, Zaslona Z (2018). Itaconate is an anti-inflammatory metabolite that activates Nrf2 via alkylation of KEAP1. Nature..

[22] Hu K, Gong X, Ai Q, Lin L, Dai J, Cai L (2017). Endogenous AMPK acts as a detrimental factor in fulminant hepatitis via potentiating JNK-dependent hepatocyte apoptosis. Cell Death Dis.

[23] Yi Z, Deng M, Scott MJ, Fu G, Loughran PA, Lei Z (2020). Immune-responsive gene 1/itaconate activates nuclear factor erythroid 2-related factor 2 in hepatocytes to protect against liver ischemia-reperfusion injury. Hepatology..

[24] Tan B, Malu S, Roth KD (2020). Development of ion pairing LC-MS/MS method for itaconate and cis-aconitate in cell extract and cell media. J Chromatogr B Anal Technol Biomed Life Sci.

[25] Peace CG, O’Neill LA. The role of itaconate in host defense and inflammation. J Clin Invest. 2022;132:e148548. 10.1172/JCI148548 PMC8759771 35040439

[26] Saeedi Saravi SS, Eroglu E, Waldeck-Weiermair M, Sorrentino A, Steinhorn B, Belousov V (2020). Differential endothelial signaling responses elicited by chemogenetic H_2_O_2_ synthesis. Redox Biol.

[27] Lee DH, Jung YS, Yun J, Han SB, Roh YS, Song MJ (2020). Peroxiredoxin 6 mediates acetaminophen-induced hepatocyte death through JNK activation. Redox Biol.

[28] Castro LS, Kviecinski MR, Ourique F, Parisotto EB, Grinevicius VM, Correia JF (2016). Albendazole as a promising molecule for tumor control. Redox Biol.

[29] Chao MR, Evans MD, Hu CW, Ji Y, Moller P, Rossner P (2021). Biomarkers of nucleic acid oxidation: a summary state-of-the-art. Redox Biol.

[30] Pang Y, Wu D, Ma Y, Cao Y, Liu Q, Tang M (2021). Reactive oxygen species trigger NF-kappaB-mediated NLRP3 inflammasome activation involvement in low-dose CdTe QDs exposure-induced hepatotoxicity. Redox Biol.

[31] Jaiswal AK, Yadav J, Makhija S, Mazumder S, Mitra AK, Suryawanshi A, et al. Irg1/itaconate metabolic pathway is a crucial determinant of dendritic cells immune-priming function and contributes to resolute allergen-induced airway inflammation. Mucosal Immunol. 2022;15:301–13. 10.1038/s41385-021-00462-y PMC8866123 34671116

[32] Ogger PP, Albers GJ, Hewitt RJ, O’Sullivan BJ, Powell JE, Calamita E, et al. Itaconate controls the severity of pulmonary fibrosis. Sci Immunol. 2020;5:eabc1884. 10.1126/sciimmunol.abc1884 PMC7116646 33097591

[33] Muri J, Wolleb H, Broz P, Carreira EM, Kopf M (2020). Electrophilic Nrf2 activators and itaconate inhibit inflammation at low dose and promote IL-1beta production and inflammatory apoptosis at high dose. Redox Biol.

[34] Okuyama H, Nakamura H, Shimahara Y, Araya S, Kawada N, Yamaoka Y (2003). Overexpression of thioredoxin prevents acute hepatitis caused by thioacetamide or lipopolysaccharide in mice. Hepatology..

[35] Wang H, Xu DX, Lu JW, Zhao L, Zhang C, Wei W (2007). N-acetylcysteine attenuates lipopolysaccharide-induced apoptotic liver damage in D-galactosamine-sensitized mice. Acta Pharm Sin.

[36] Lyu H, Wang H, Li L, Zhu J, Chen F, Chen Y (2020). Hepatocyte-specific deficiency of Nrf2 exacerbates carbon tetrachloride-induced liver fibrosis via aggravated hepatocyte injury and subsequent inflammatory and fibrogenic responses. Free Radic Biol Med.

[37] Silva-Gomes S, Santos AG, Caldas C, Silva CM, Neves JV, Lopes J (2014). Transcription factor NRF2 protects mice against dietary iron-induced liver injury by preventing hepatocytic cell death. J Hepatol.

[38] Mohs A, Otto T, Schneider KM, Peltzer M, Boekschoten M, Holland CH (2021). Hepatocyte-specific NRF2 activation controls fibrogenesis and carcinogenesis in steatohepatitis. J Hepatol.

[39] Michelucci A, Cordes T, Ghelfi J, Pailot A, Reiling N, Goldmann O (2013). Immune-responsive gene 1 protein links metabolism to immunity by catalyzing itaconic acid production. Proc Natl Acad Sci USA.

[40] Daniels BP, Kofman SB, Smith JR, Norris GT, Snyder AG, Kolb JP (2019). The nucleotide sensor ZBP1 and kinase RIPK3 induce the enzyme IRG1 to promote an antiviral metabolic state in neurons. Immunity..

[41] Cai L, Huang J, Huang D, Lv H, Wang D, Wang H (2023). Deficiency of immune-responsive gene 1 exacerbates interleukin-1beta-elicited the inflammatory response of chondrocytes via enhancing the activation of NLRP3 inflammasome. Int Immunopharmacol.

[42] Pan J, Zhao X, Lin C, Xu H, Yin Z, Liu T (2014). Immune responsive gene 1, a novel oncogene, increases the growth and tumorigenicity of glioma. Oncol Rep.

[43] Benhar M. Oxidants, antioxidants and thiol redox switches in the control of regulated cell death pathways. Antioxidants. 2020;9:309. 10.3390/antiox9040309 PMC7222211 32290499

[44] Moldogazieva NT, Lutsenko SV, Terentiev AA (2018). Reactive oxygen and nitrogen species-induced protein modifications: implication in carcinogenesis and anticancer therapy. Cancer Res.

[45] Peng X, Yang Y, Tang L, Wan J, Dai J, Li L (2020). Therapeutic benefits of apocynin in mice with lipopolysaccharide/D-galactosamine-induced acute liver injury via suppression of the late stage pro-apoptotic AMPK/JNK pathway. Biomed Pharmacother.

[46] Chen MB, Wu XY, Gu JH, Guo QT, Shen WX, Lu PH (2011). Activation of AMP-activated protein kinase contributes to doxorubicin-induced cell death and apoptosis in cultured myocardial H9c2 cells. Cell Biochem Biophys.

[47] Sook SH, Lee HJ, Kim JH, Sohn EJ, Jung JH, Kim B (2014). Reactive oxygen species-mediated activation of AMP-activated protein kinase and c-Jun N-terminal kinase plays a critical role in beta-sitosterol-induced apoptosis in multiple myeloma U266 cells. Phytother Res.

[48] Cai Y, Martens GA, Hinke SA, Heimberg H, Pipeleers D, Van de Casteele M (2007). Increased oxygen radical formation and mitochondrial dysfunction mediate beta cell apoptosis under conditions of AMP-activated protein kinase stimulation. Free Radic Biol Med.

[49] Zmijewski JW, Banerjee S, Bae H, Friggeri A, Lazarowski ER, Abraham E (2010). Exposure to hydrogen peroxide induces oxidation and activation of AMP-activated protein kinase. J Biol Chem.

[50] Ren Y, Shen HM (2019). Critical role of AMPK in redox regulation under glucose starvation. Redox Biol.

[51] Huang DD, Yan XL, Fan SD, Chen XY, Yan JY, Dong QT (2020). Nrf2 deficiency promotes the increasing trend of autophagy during aging in skeletal muscle: a potential mechanism for the development of sarcopenia. Aging.

[52] Jia Y, Wang H, Wang Q, Ding H, Wu H, Pan H (2016). Silencing Nrf2 impairs glioma cell proliferation via AMPK-activated mTOR inhibition. Biochem Biophys Res Commun.

[53] Che Q, Lin L, Ai Q, Ge P, Dai J, Jiang R (2015). Caloric restriction mimetic 2-deoxyglucose alleviated lethal liver injury induced by lipopolysaccharide/D-galactosamine in mice. Biochem Biophys Res Commun.

[54] Runtsch MC, Angiari S, Hooftman A, Wadhwa R, Zhang Y, Zheng Y (2022). Itaconate and itaconate derivatives target JAK1 to suppress alternative activation of macrophages. Cell Metab.

[55] Hooftman A, Angiari S, Hester S, Corcoran SE, Runtsch MC, Ling C (2020). The immunomodulatory metabolite itaconate modifies NLRP3 and inhibits inflammasome activation. Cell Metab.

[56] Zhang Z, Chen C, Yang F, Zeng YX, Sun P, Liu P (2022). Itaconate is a lysosomal inducer that promotes antibacterial innate immunity. Mol Cell.

[57] Strelko CL, Lu W, Dufort FJ, Seyfried TN, Chiles TC, Rabinowitz JD (2011). Itaconic acid is a mammalian metabolite induced during macrophage activation. J Am Chem Soc.

[58] Lampropoulou V, Sergushichev A, Bambouskova M, Nair S, Vincent EE, Loginicheva E (2016). Itaconate links inhibition of succinate dehydrogenase with macrophage metabolic remodeling and regulation of inflammation. Cell Metab.

[59] Zeng YR, Song JB, Wang D, Huang ZX, Zhang C, Sun YP, et al. The immunometabolite itaconate stimulates OXGR1 to promote mucociliary clearance during the pulmonary innate immune response. J Clin Invest. 2023;133:e160463. 10.1172/JCI160463 PMC10014103 36919698

