# Supplementary material for: Negative regulation of pro-apoptotic AMPK/JNK pathway by itaconate in mice with fulminant liver injury
Source: Cell Death Dis. 2023 Jul 31;14(7):486. doi: 10.1038/s41419-023-06001-w (PMC10390640; doi:10.1038/s41419-023-06001-w)
Supplement: Supplementary file 1 — supplementary information [file 41419_2023_6001_MOESM1_ESM.docx]

**Supplementary methods**

**Identification of the genotype of IRG1 KO mice and Hepatocyte-specific Nrf2 KO mice.**

The genotype of the mice was identified by PCR analysis of toe DNA using the following primers: Irg1^-/-^ homozygous mice (forward: 5′-3′: CTC CAA TCT GAC ACG TCT TCT AG and reverse: 5′-3′: AGA GGA AGA TGA AGT GGG AGA ATT); wild-type mice (forward: 5′-3′: TGT TAC AGT CAG AGA TGG AGA GG and reverse: 5′-3′: TGT GTC AGG TAC GGT AAT GAG TG); Nrf2 floxed (Nrf2^fl/fl^) mice (forward: 5′-3′: TCA TGA GAG CTT CCC AGA CTC and reverse: 5′-3′: CAG CCA GCT GCT TGT TTT C) and Alb-Cre PCR (forward: 5′-3′: GAA GCA GAA GCT TAG GAA GAT GG and reverse: 5′-3′: TTG GCC CCT TAC CAT AAC TG).

**Supplementary experimental protocol**

To investigate the potential association of AMPK with the exacerbated liver injury in IRG1 KO mice, vehicle or the AMPK inhibitor compound C (15 mg/kg, dissolved in DMSO) was administered intraperitoneally in LPS/D-Gal-challenged IRG1 KO mice. The animals were sacrificed at 6 h after LPS/D-Gal injection, the liver samples and serum samples were collected for further experiments.

To investigate the potential association of JNK with the exacerbated liver injury in IRG1 KO mice, vehicle or the JNK inhibitor SP600125 (50 mg/kg, dissolved in DMSO) was administered intraperitoneally in LPS/D-Gal-challenged IRG1 KO mice. The animals were sacrificed at 6 h after LPS/D-Gal injection, the liver samples and serum samples were collected for further experiments.

To investigate the potential association of oxidative stress with the exacerbated liver injury in IRG1 KO mice, vehicle or N-acetylcysteine (NAC, 120 mg/kg, dissolved in normal saline) was administered intraperitoneally in LPS/D-Gal-challenged IRG1 KO mice. The animals were sacrificed at 6 h after LPS/D-Gal injection, the liver samples and serum samples were collected for further experiments.

To investigate the potential effects of 4-octyl itaconate (4-OI) on LPS/D-Gal-induced liver injury in C57BL/6 mice without IRG1 deletion, 4-OI was administered in mice 0.5 h before LPS/D-Gal exposure and the animals were sacrificed 6 h post LPS/D-Gal exposure. The liver samples and serum samples were collected for further experiments.

**Determination of the pro-inflammatory cytokines**

Enzyme-linked immunosorbent assays (ELISAs) were performed to detect the serum concentrations of cytokines (TNF-α and IL-6) via ELISA kit (Neobioscience, China), according to the manufacturer's protocols.

**Figure legends**

**Supplementary Figure 1 Genetic deletion of IRG1 in mice.**

(A) Schematic representation of the strategy used to produce IRG1 KO mice. (B) The genotype of wild-type (WT) and IRG1 knockout (KO) mice. (C) The mRNA level of IRG1 in liver from WT and IRG1KO mice. (D) The level of IRG1 protein was determined (n=4). All data were expressed as mean ± SD.

**Supplementary Figure 2 IRG1 deficiency enhanced LPS/D-Gal-induced production of pro-inflammatory cytokines.**

WT or IRG1 KO mice with fulminant liver injury were sacrificed 6 h post LPS/D-Gal exposure. (A) The serum TNF-α level was detected (n=8). (B) The serum IL-6 level was detected (n=8). All data were expressed as mean ± SD. All data were expressed as mean ± SD.

**Supplementary Figure 3 Inhibition of AMPK reversed the aggravated liver injury in IRG1 KO mice.**

IRG1 KO mice with fulminant liver injury were supplemented with the AMPK inhibitor compound C and sacrificed 6 h post LPS/D-Gal exposure. (A) The hepatic levels of phosphorylated AMPK (pAMPK), total AMPK (AMPK), phosphorylated JNK (pJNK), total JNK (JNK) and cleaved caspase-3 were determined (n=4). (B) The hepatic activities of caspase-8, caspase-9 and caspase-3 were determined (n=8). (C) The TUNEL-positive cells were counted. (D) The serum level of ALT and AST were determined (n=8). (E) The liver sections were stained with hematoxylin & eosin for histological examination (scale bar: 100 μm). All data were expressed as mean ± SD.

**Supplementary Figure 4 Inhibition of JNK reversed the aggravated liver injury in IRG1 KO mice.**

IRG1 KO mice with fulminant liver injury were supplemented with the JNK inhibitor SP600125 and sacrificed 6 h post LPS/D-Gal exposure. (A) The hepatic protein levels of pJNK, JNK and cleaved caspase-3 were detected (n=4). (B) The hepatic activities of caspase-8, caspase-9 and caspase-3 were determined (n=8). (C) The TUNEL-positive cells were counted. (D) The serum level of ALT and AST were determined (n=8). (E) The liver sections were stained with hematoxylin & eosin for histological examination (scale bar: 100 μm). All data were expressed as mean ± SD.

**Supplementary Figure 5 Treatment with 4-octyl itaconate did not induce liver injury or oxidative stress.**

4-octyl itaconate (4-OI) was administered in C57BL/6 mice with fulminant liver injury 1.5 h post LPS/D-Gal exposure and the animals were sacrificed 6 h post LPS/D-Gal exposure. (A) The hepatic activities of caspase-3 were determined (n=8). (B) The serum level of ALT and AST were determined (n=8). (C) The hepatic contents of TBARS, the ratio of GSSG/GSH in liver and the serum level of 8-OH-dG were determined (n=8). All data were expressed as mean ± SD.

**Supplementary Figure 6 Hepatocyte-specific deletion of Nrf2 in mice.**

(A) Schematic representation of the strategy used to produce hepatocyte-specific Nrf2^-/-^ (Nrf2^LKO^) mice by backcrossing Nrf2^fl/fl^ and Alb-Cre mice. (B) The genotype of Nrf2^fl/fl^ and Nrf2^LKO^ mice. (C) The protein level of Nrf2 in liver from LPS/D-Gal-challenged Nrf2^fl/fl^ and Nrf2^LKO^ mice. (D) The serum level of ALT and AST were determined (n=8). (E) The liver sections were stained with hematoxylin & eosin for histological examination (scale bar: 100 μm). All data were expressed as mean ± SD.

**Supplementary Figure 7 Treatment with the antioxidant NAC reversed the aggravated liver injury in IRG1 KO mice.**

IRG1 KO mice with fulminant liver injury were supplemented with N-acetylcysteine (NAC) and sacrificed 6 h post LPS/D-Gal exposure. (A) The hepatic contents of TBARS, the ratio of GSSG/GSH in liver and the serum level of 8-OH-dG were determined (n=8). (B) The hepatic levels of phosphorylated AMPK (pAMPK), total AMPK (AMPK), phosphorylated JNK (pJNK), total JNK (JNK) and cleaved caspase-3 were determined (n=4). (C) The hepatic activities of caspase-8, caspase-9 and caspase-3 were determined (n=8). (D) The TUNEL-positive cells were counted. (E) The serum level of ALT and AST were determined (n=8). (F) The liver sections were stained with hematoxylin & eosin for histological examination (scale bar: 100 μm). All data were expressed as mean ± SD.
