# Supplementary figures and images for: Negative regulation of pro-apoptotic AMPK/JNK pathway by itaconate in mice with fulminant liver injury
Source: Cell Death Dis. 2023 Jul 31;14(7):486. doi: 10.1038/s41419-023-06001-w (PMC10390640; doi:10.1038/s41419-023-06001-w)

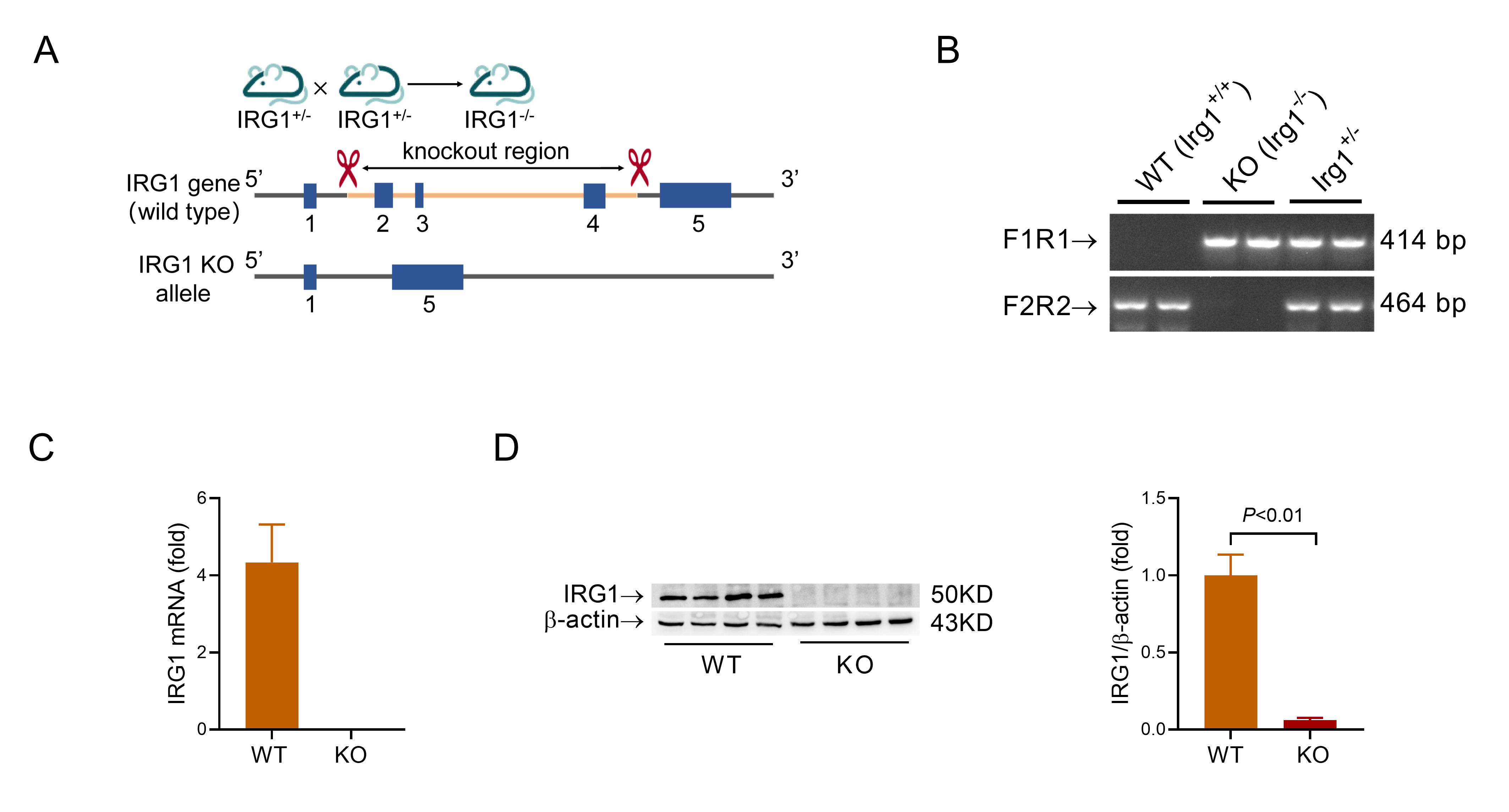

Supplement: Supplementary file 2 — Supplementary figure 1 [file 41419_2023_6001_MOESM2_ESM.tif]

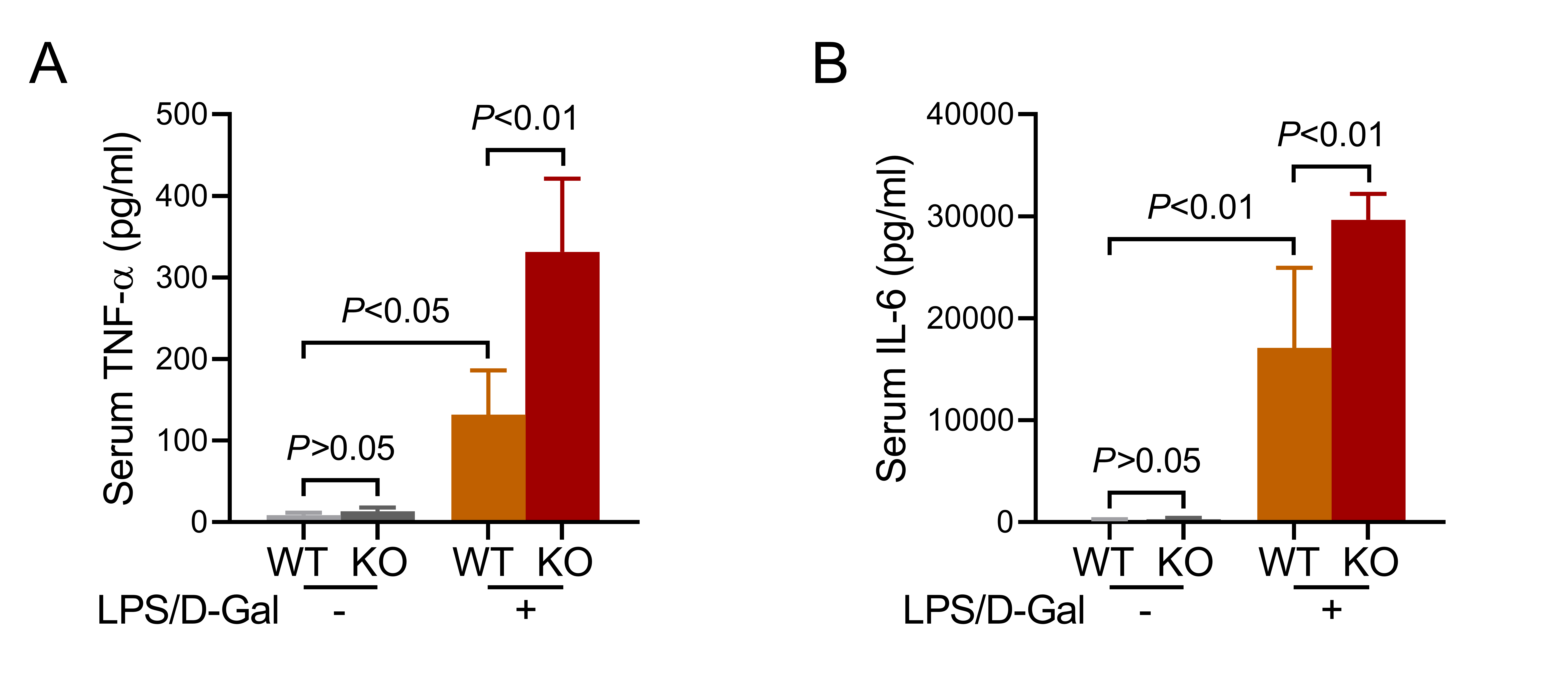

Supplement: Supplementary file 3 — Supplementary figure 2 [file 41419_2023_6001_MOESM3_ESM.tif]

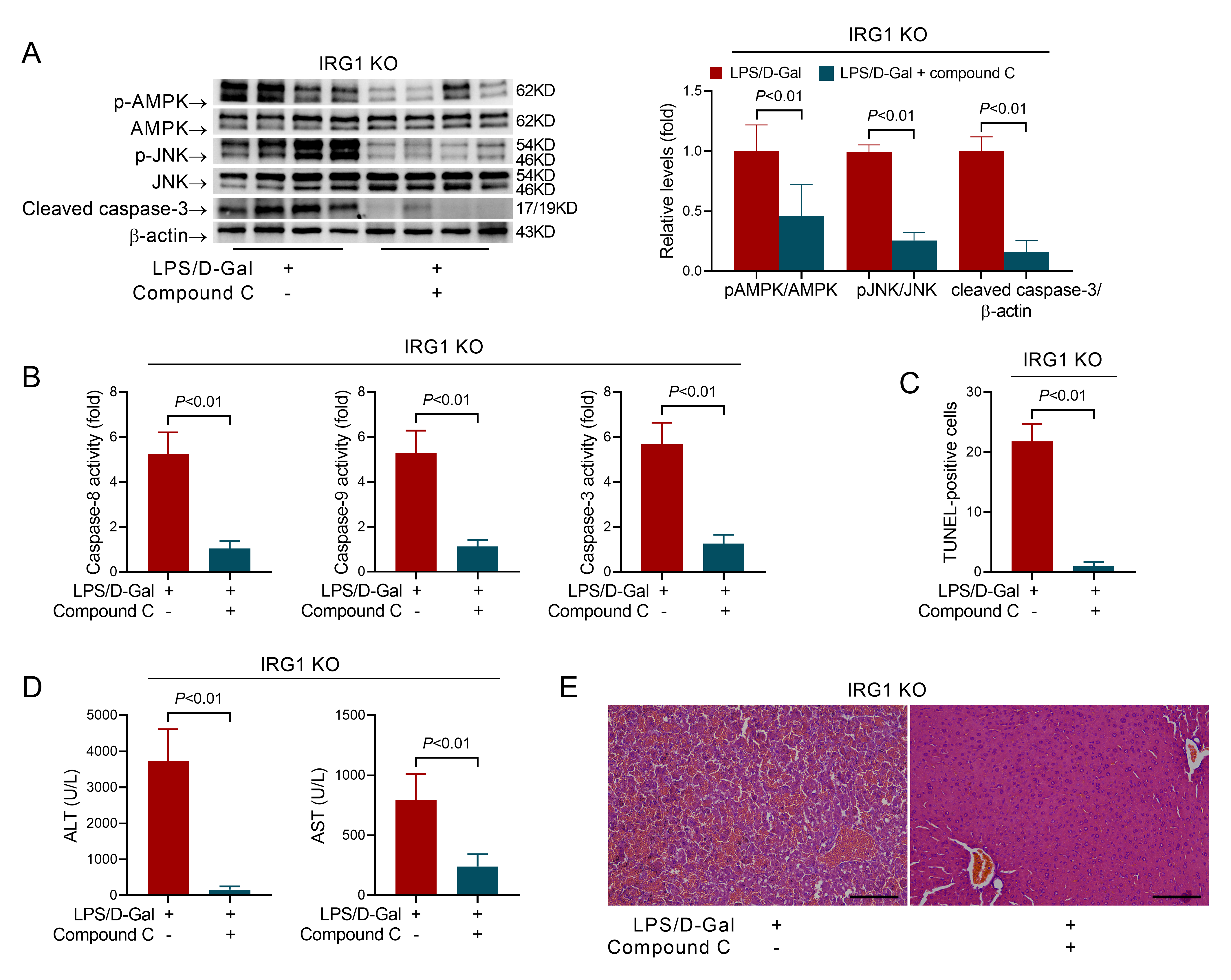

Supplement: Supplementary file 4 — Supplementary figure 3 [file 41419_2023_6001_MOESM4_ESM.tif]

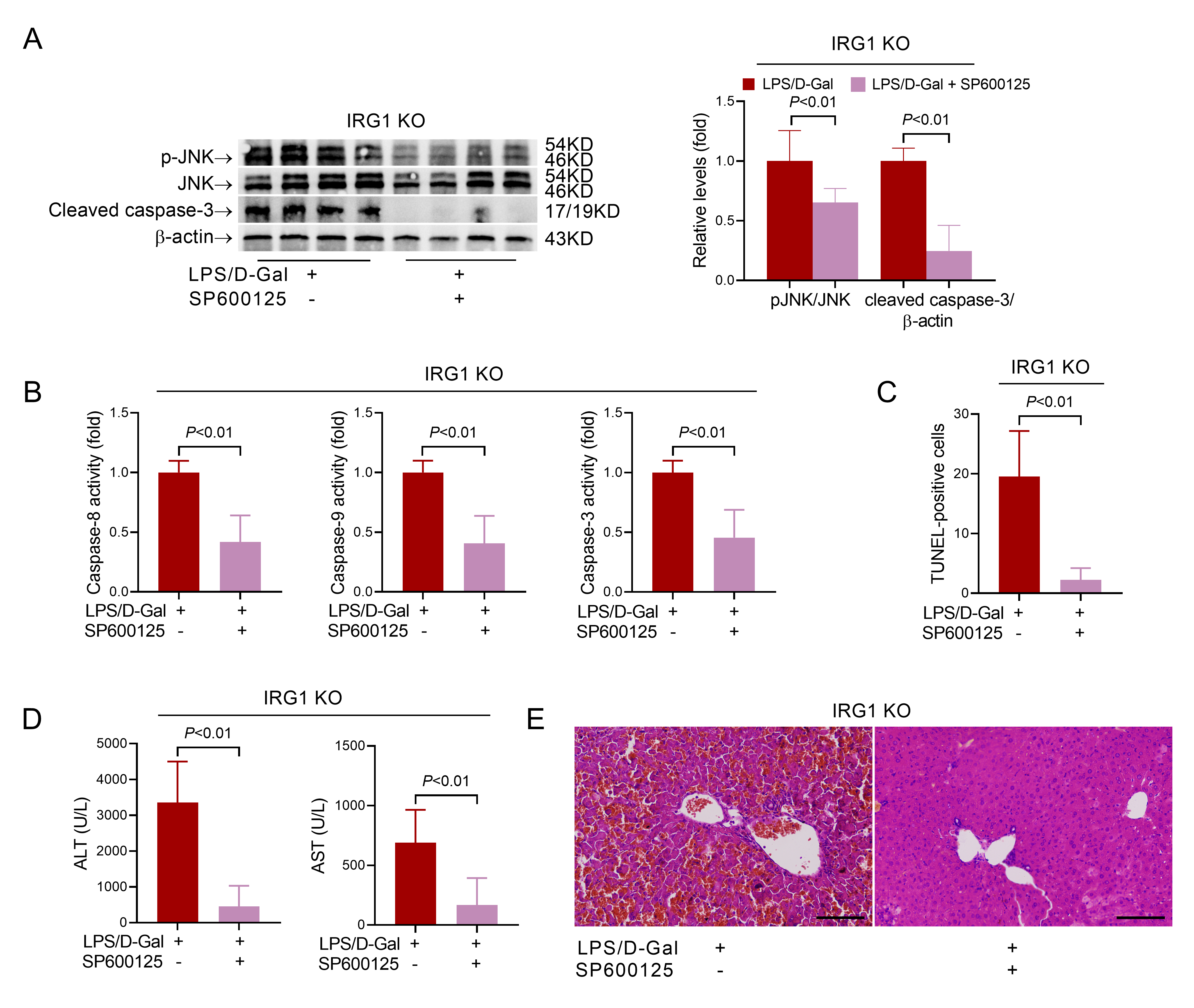

Supplement: Supplementary file 5 — Supplementary figure 4 [file 41419_2023_6001_MOESM5_ESM.tif]

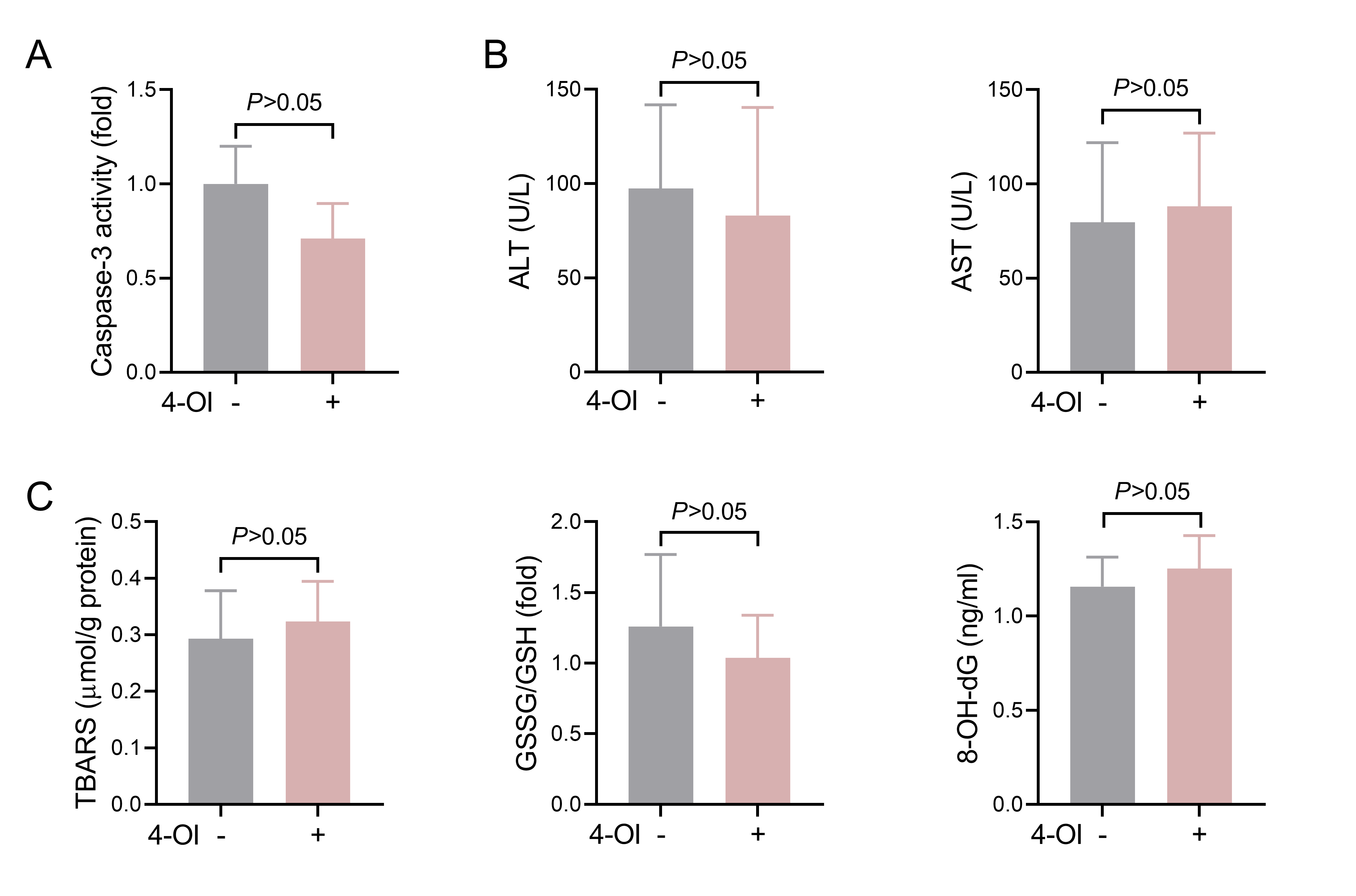

Supplement: Supplementary file 6 — Supplementary figure 5 [file 41419_2023_6001_MOESM6_ESM.tif]

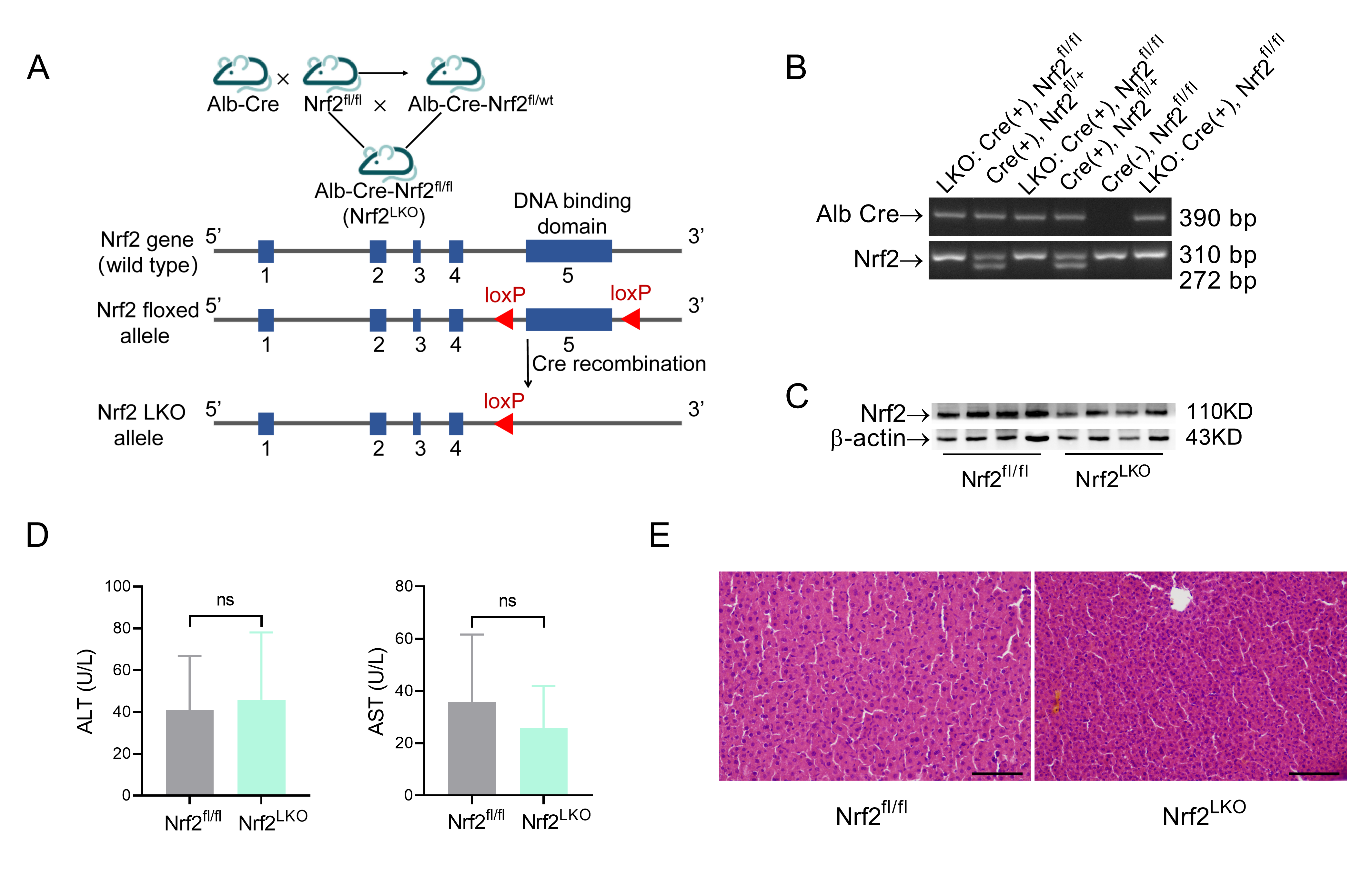

Supplement: Supplementary file 7 — Supplementary figure 6 [file 41419_2023_6001_MOESM7_ESM.tif]

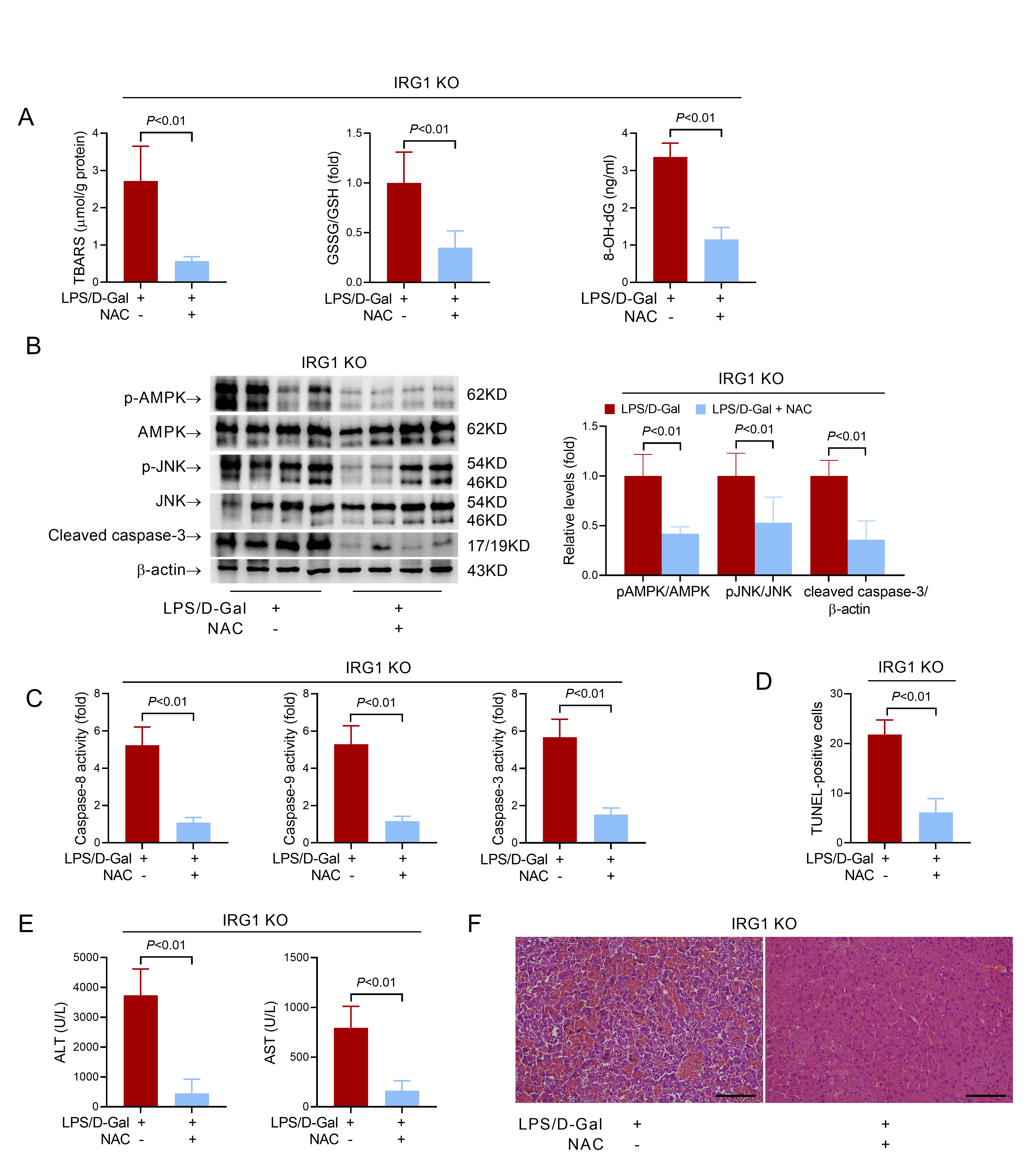

Supplement: Supplementary file 8 — Supplementary figure 7 [file 41419_2023_6001_MOESM8_ESM.tif]
