# Supplementary material for: Negative regulation of pro-apoptotic AMPK/JNK pathway by itaconate in mice with fulminant liver injury
Source: Cell Death Dis. 2023 Jul 31;14(7):486. doi: 10.1038/s41419-023-06001-w (PMC10390640; doi:10.1038/s41419-023-06001-w)
Supplement: Supplementary file 10 — Original Data File [file 41419_2023_6001_MOESM10_ESM.pdf]

Figure 1D IRG1

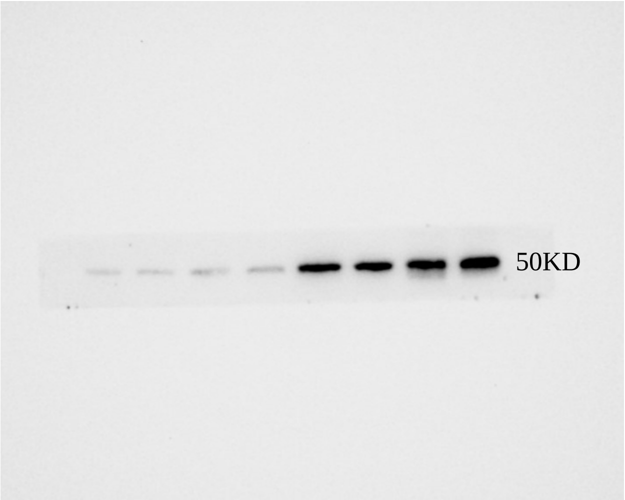

Figure 1D β-actin

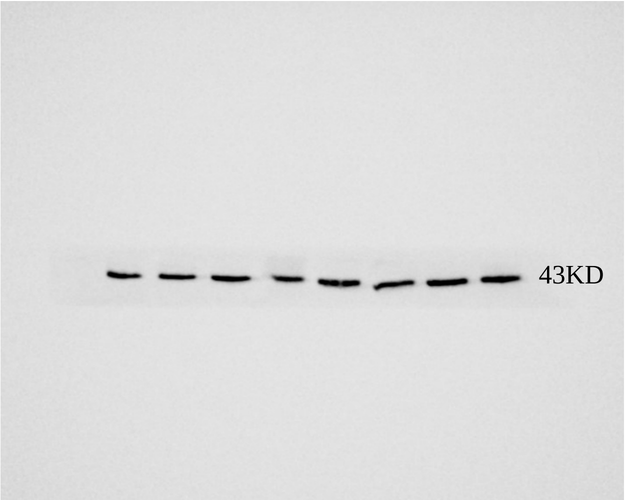

Figure 1 E IRG1

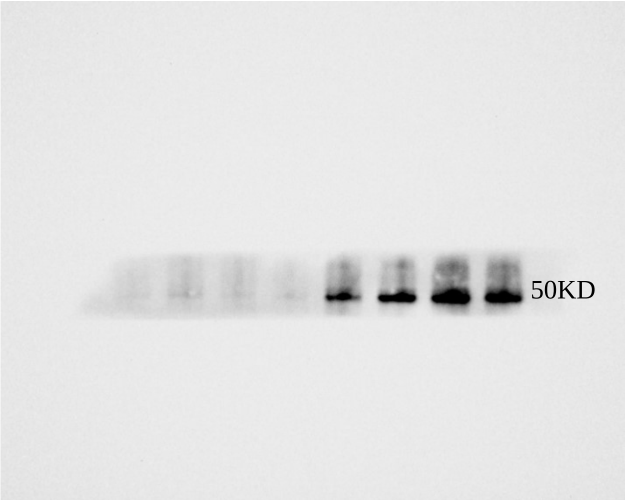

Figure 1E β-actin

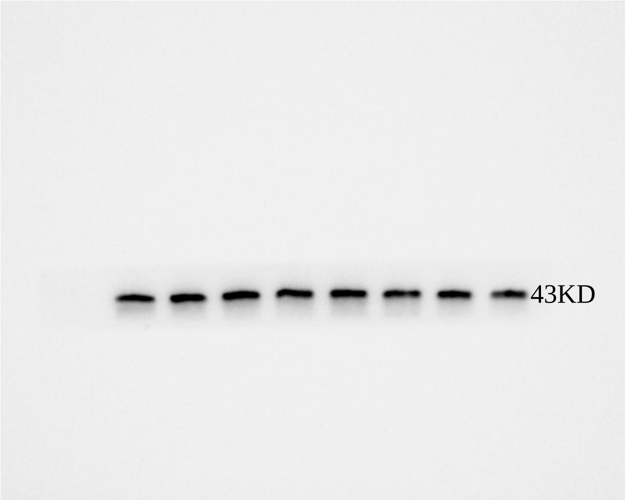

Figure 2D Cleaved caspase-3

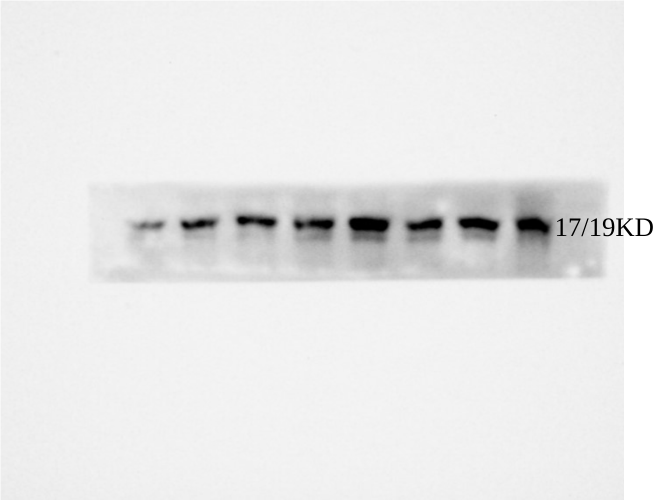

Figure 2D β-actin

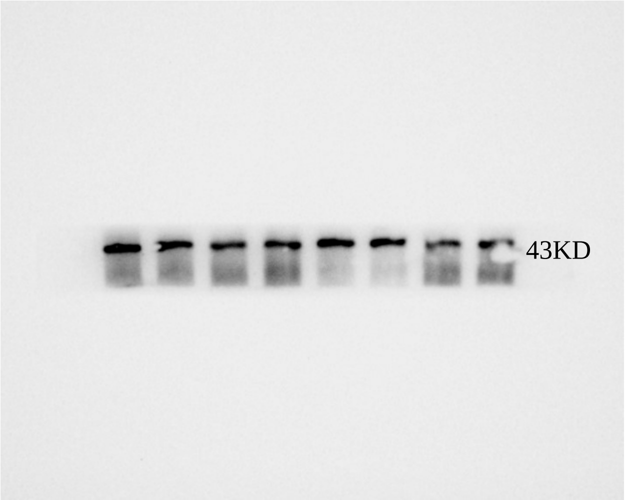

Figure 3D p-AMPK

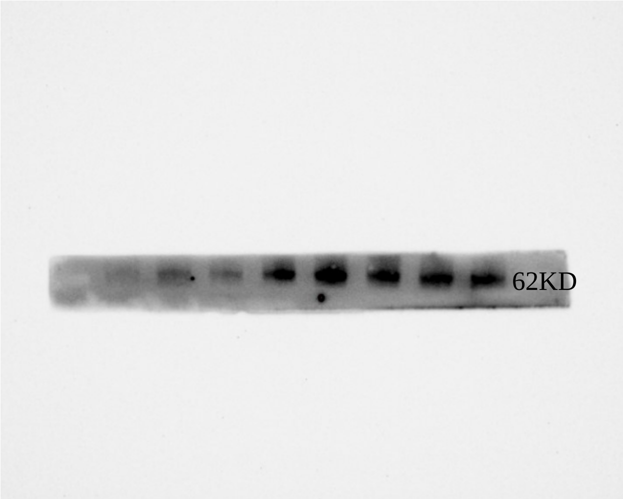

Figure 3D AMPK

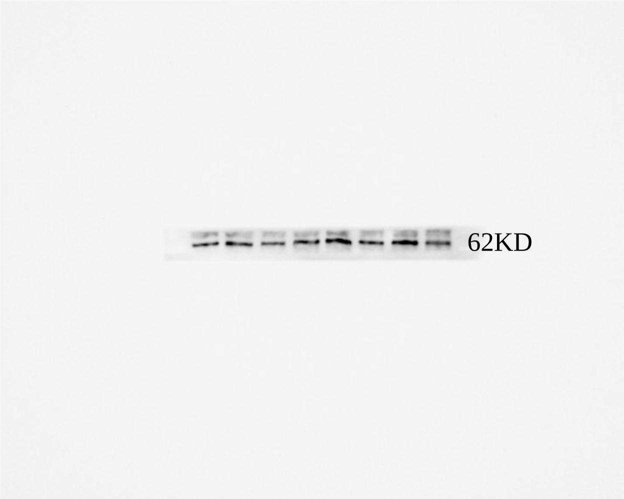

Figure 3D p-JNK

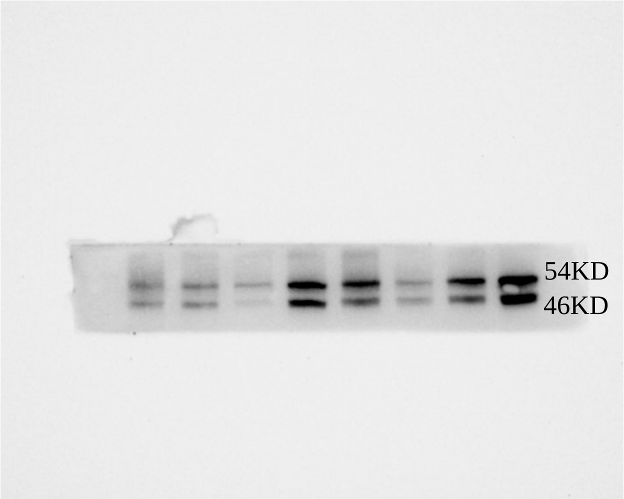

Figure 3D JNK

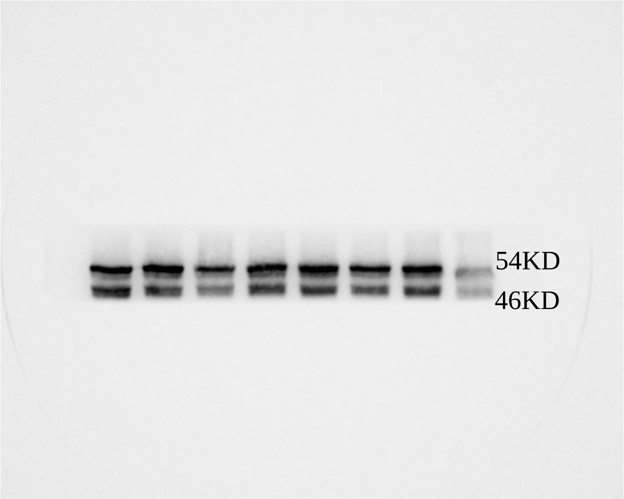

Figure 4C p-AMPK

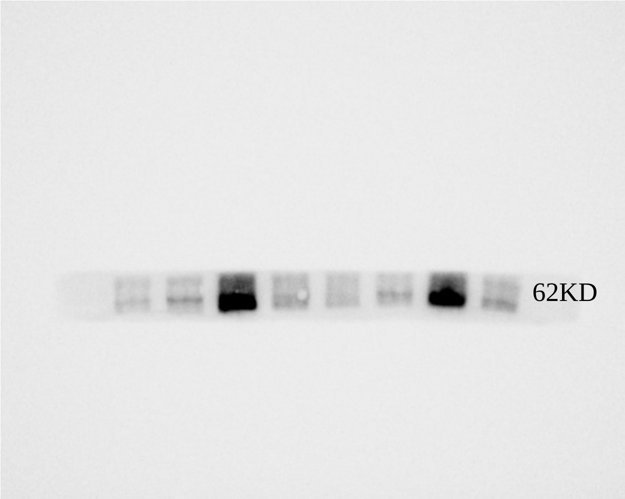

Figure 4C AMPK

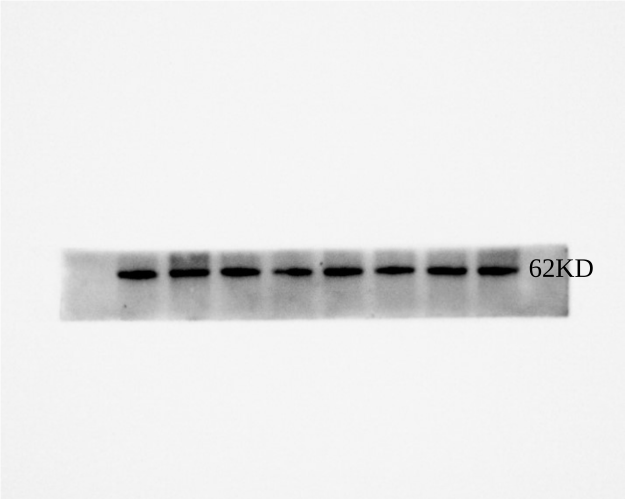

Figure 4C p-JNK

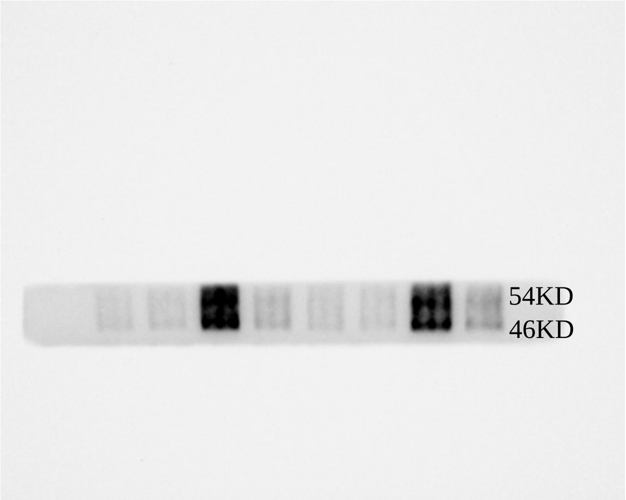

Figure 4C JNK

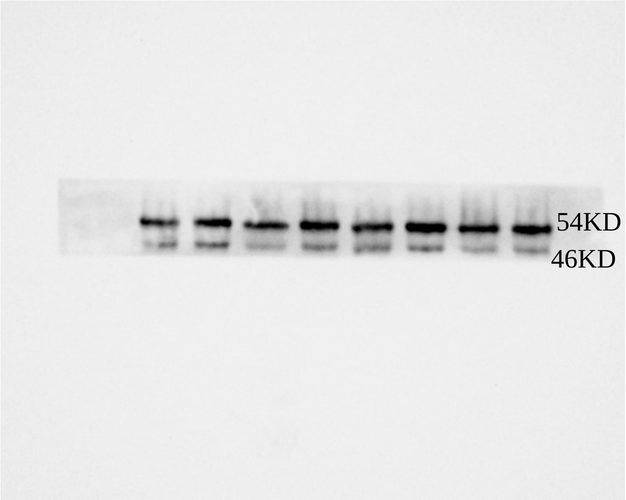

Figure 4C Cleaved caspase 3

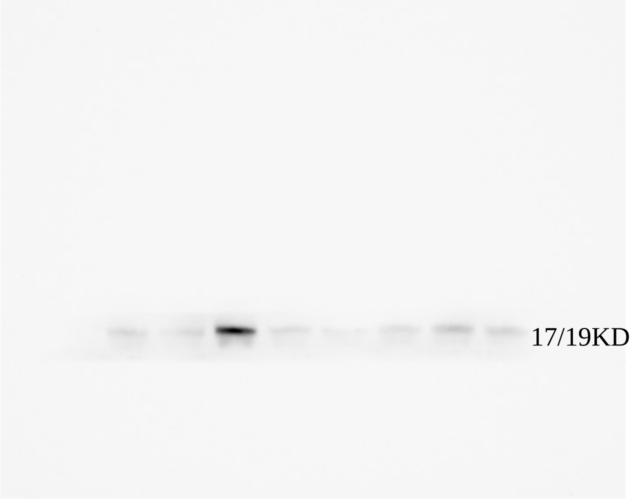

Figure 4C β-actin

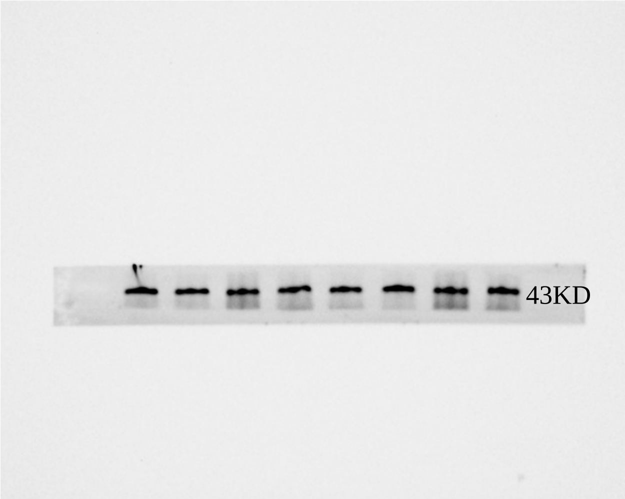

Figure 4C p-AMPK

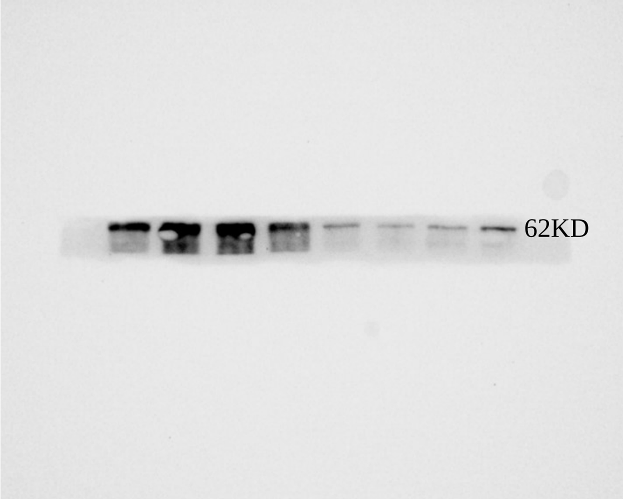

Figure 4C AMPK

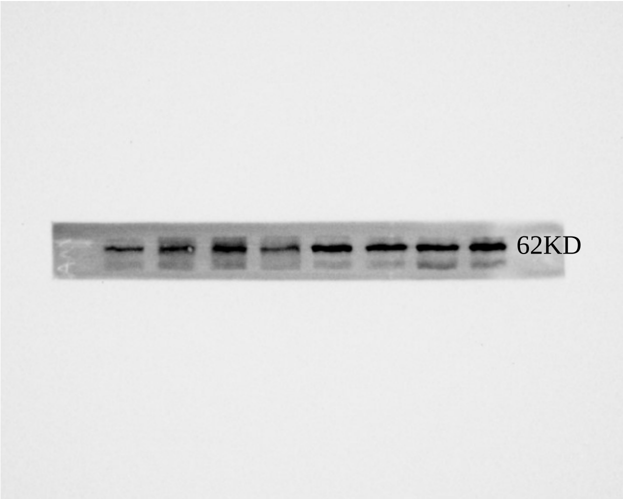

Figure 4C p-JNK

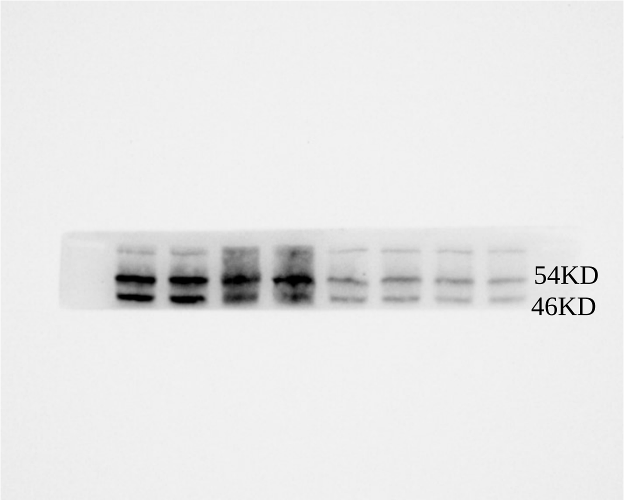

Figure 4C JNK

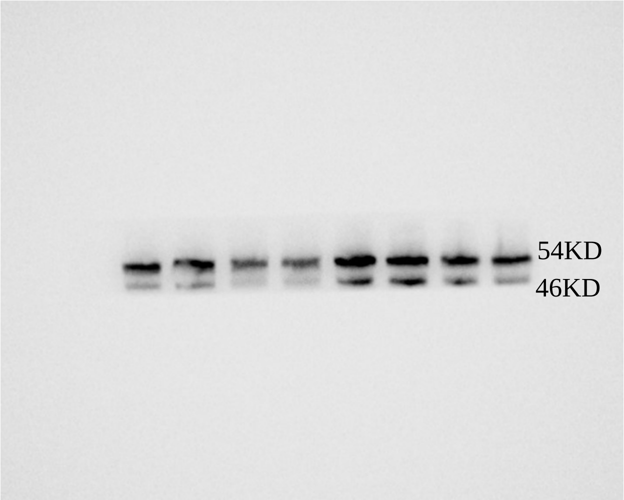

Figure 4C Cleaved caspase-3

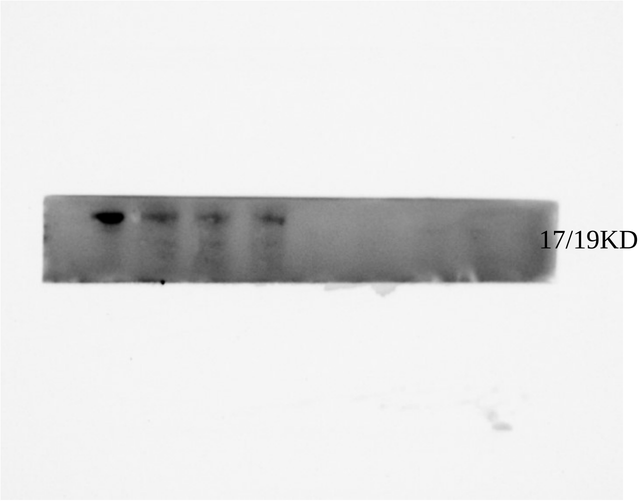

Figure 4C β-actin

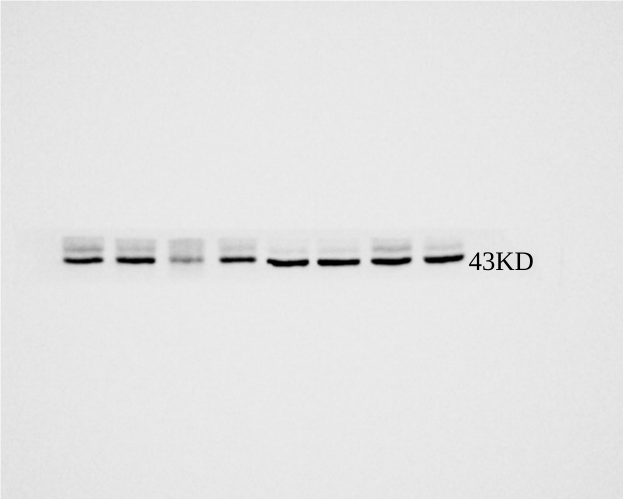

Figure 5A p-AMPK

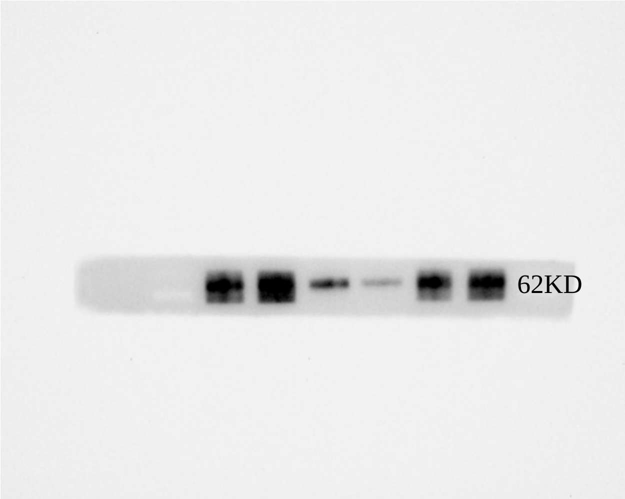

Figure 5A AMPK

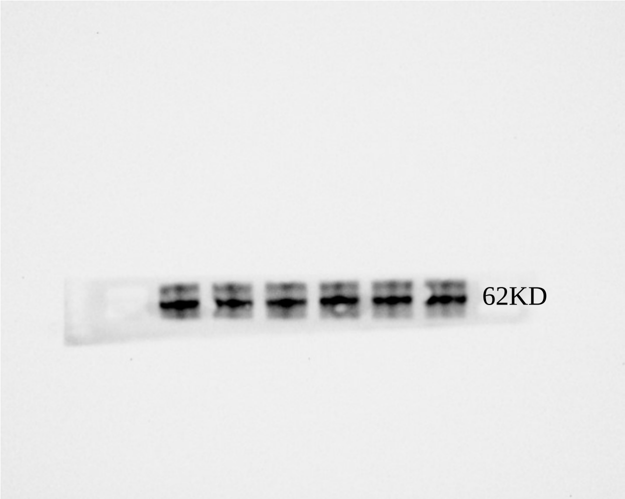

Figure 5A p-JNK

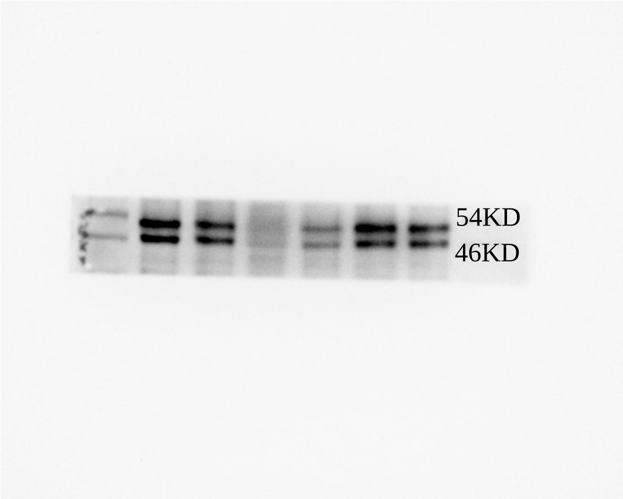

Figure 5A JNK

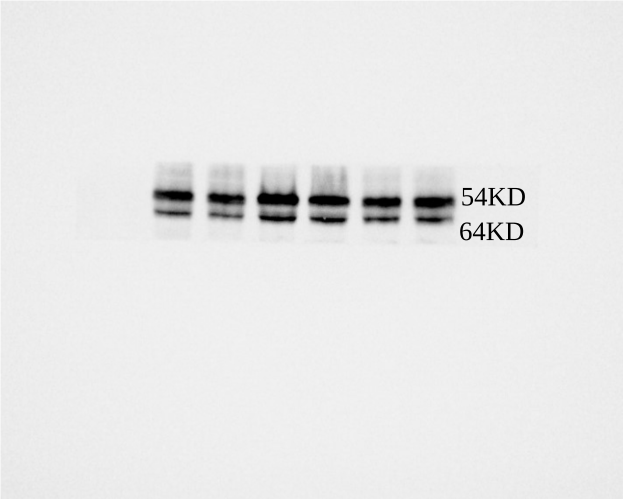

Figure 5A Cleaved caspase-3

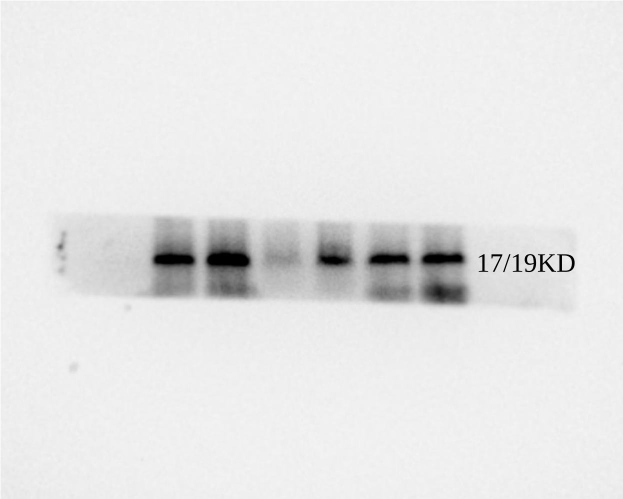

Figure 5A β-actin

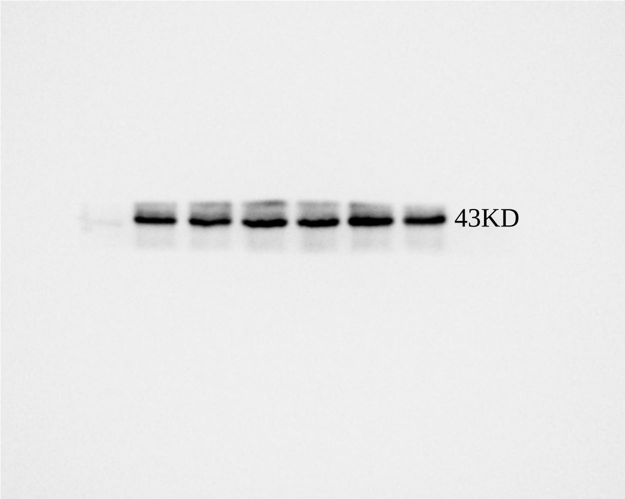

Figure 6B Nrf2

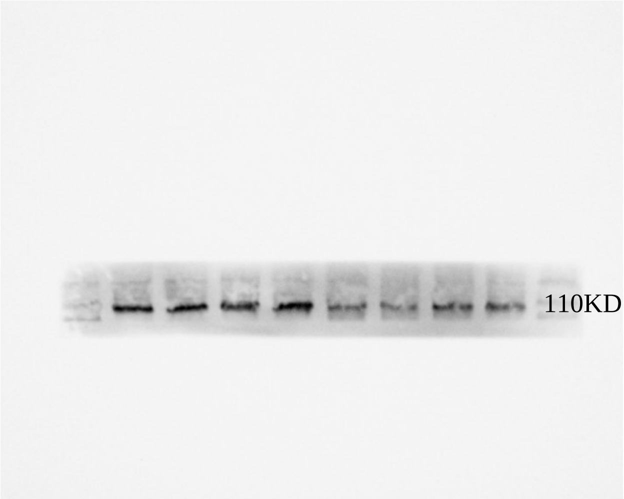

Figure 6B HO-1

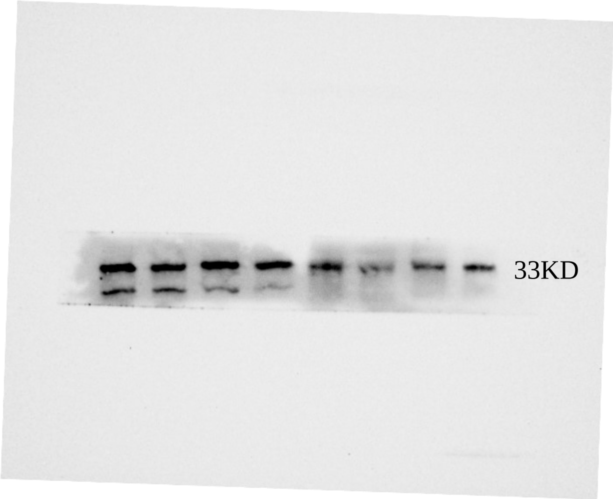

Figure 6B NQO1

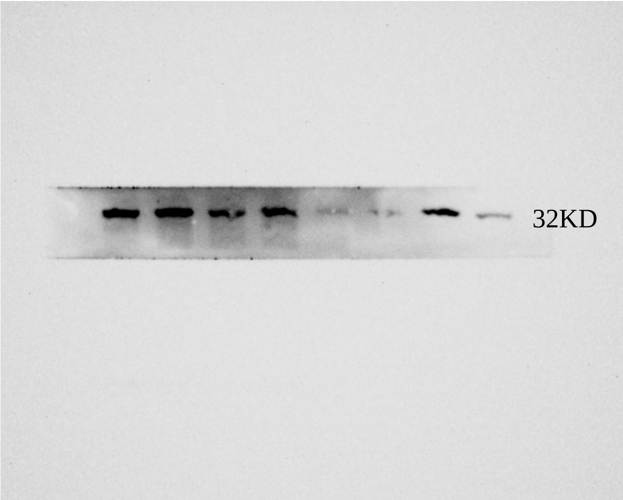

Figure 6B β-actin

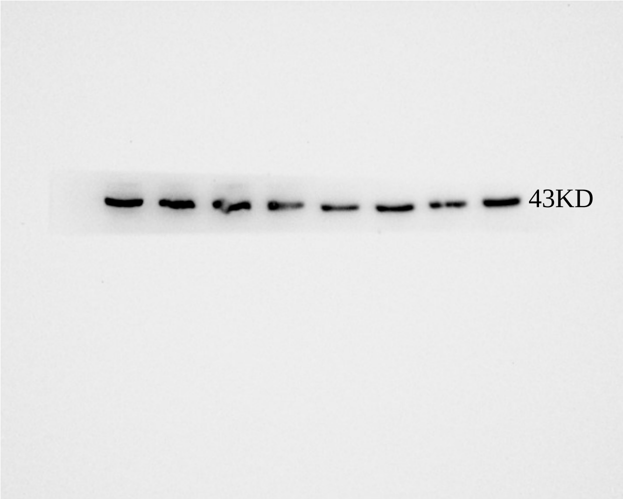

Figure 6D Nrf2

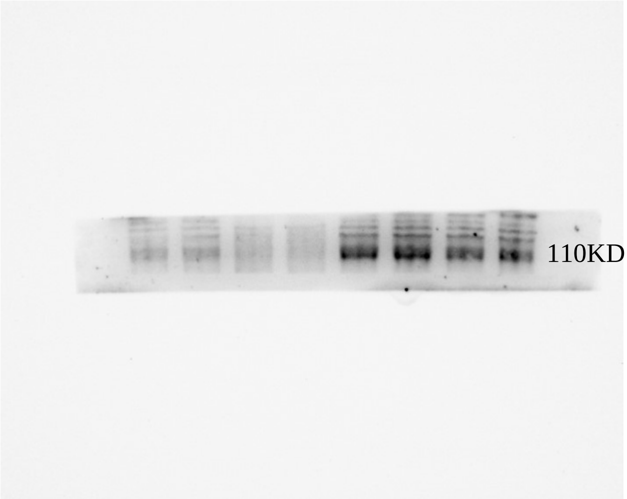

Figure 6D HO-1

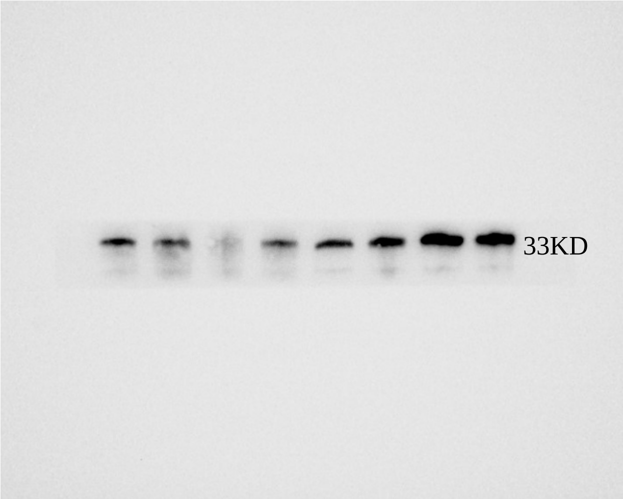

Figure 6D NQO1 t

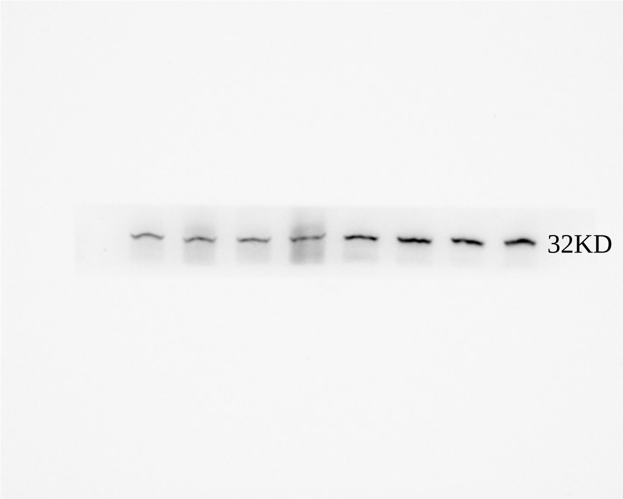

Figure 6D β-actin

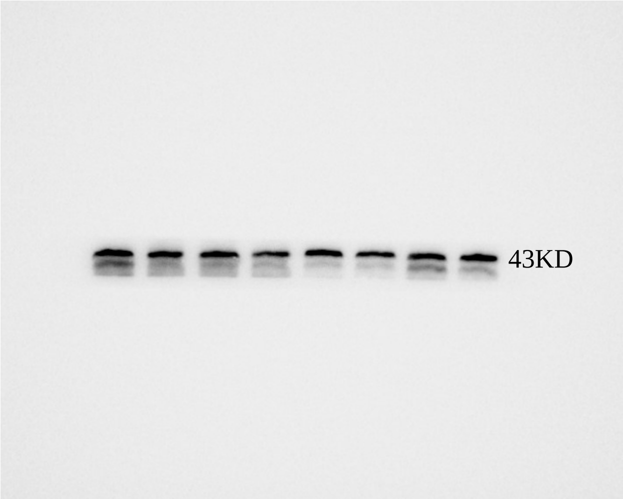

Figure 7D p-AMPK

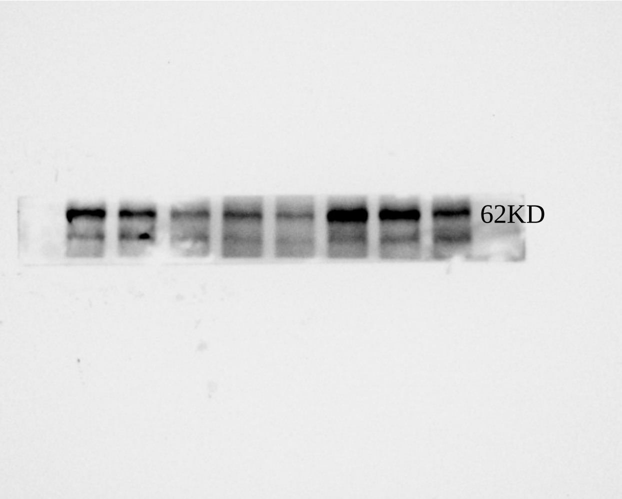

Figure 7D AMPK

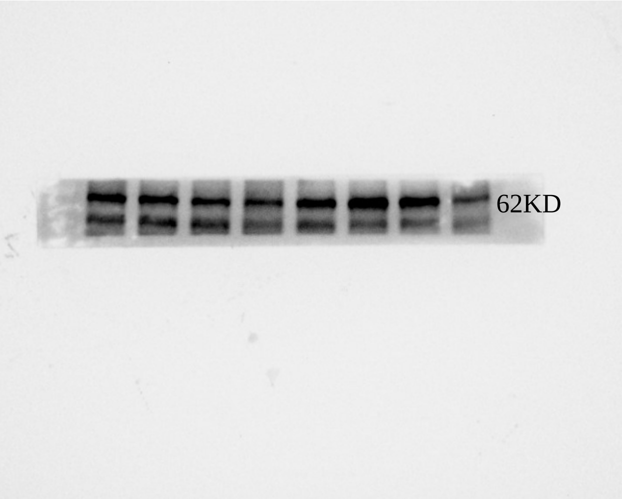

Figure 7D p-JNK

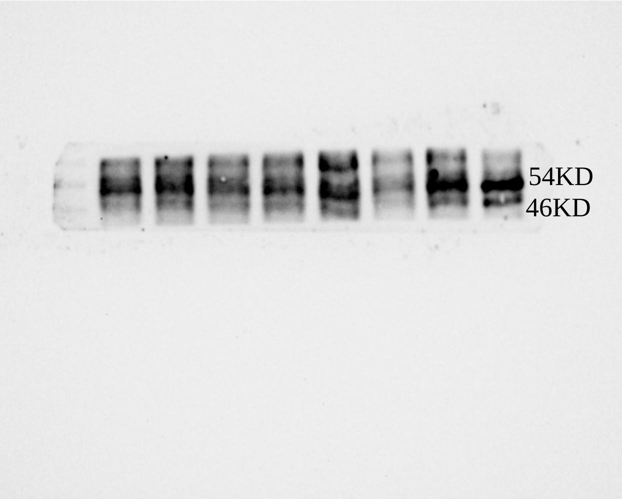

Figure 7D JNK

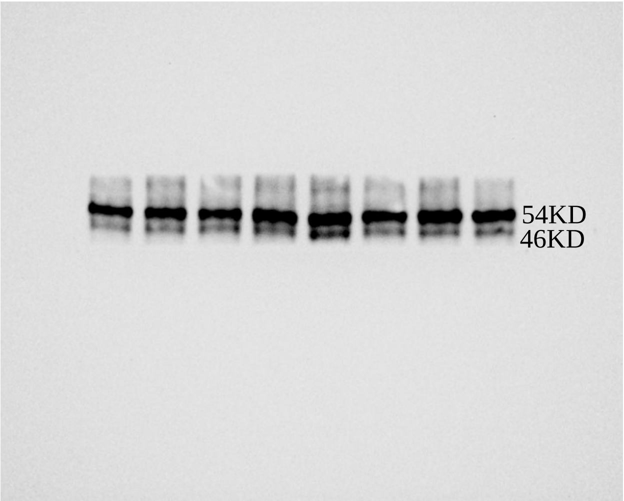

Figure 8A Nrf2

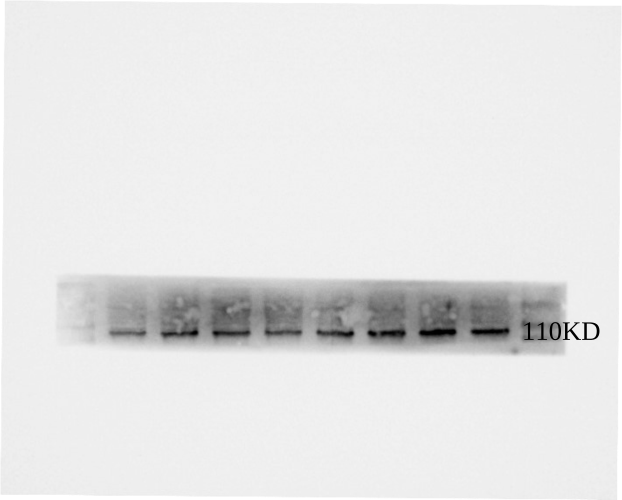

Figure 8A HO-1

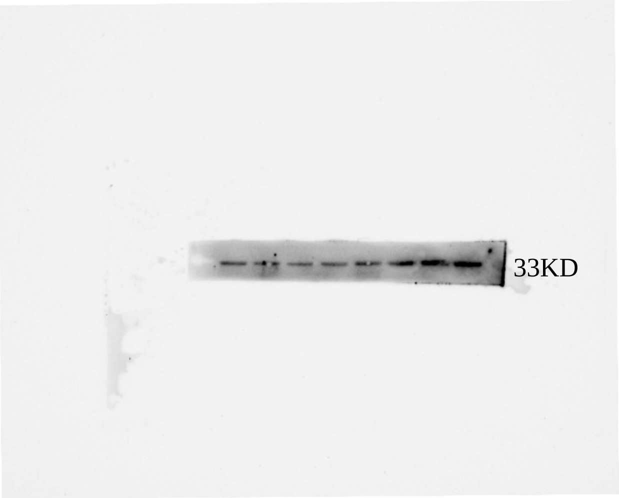

Figure 8A NQO1

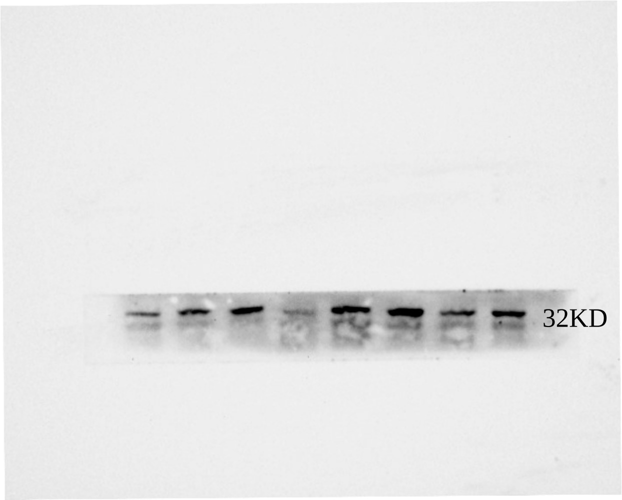

Figure 8A β-actin

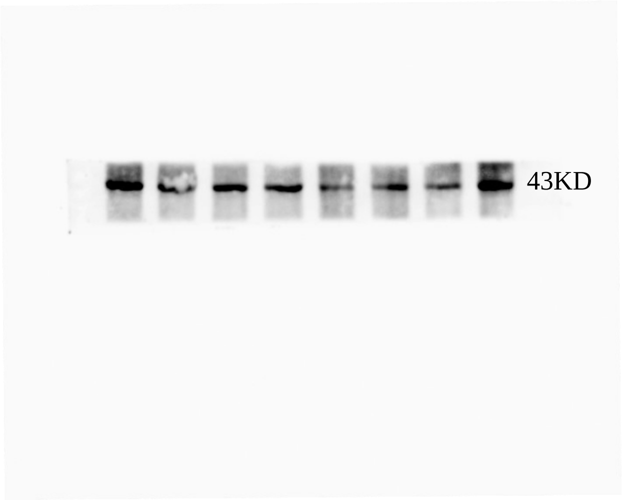

Figure 8C p-AMPK

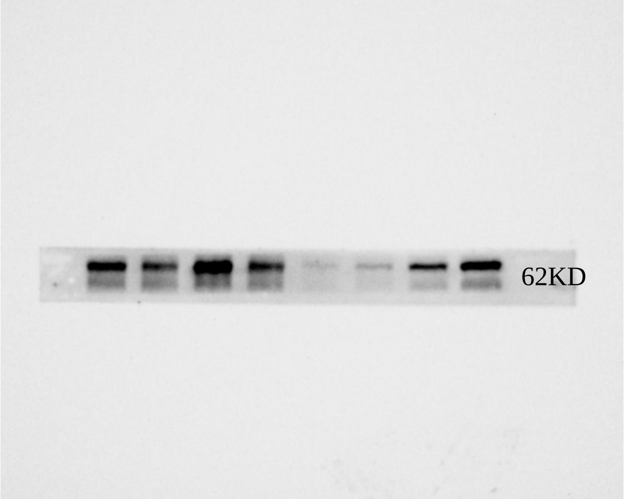

Figure 8C AMPK

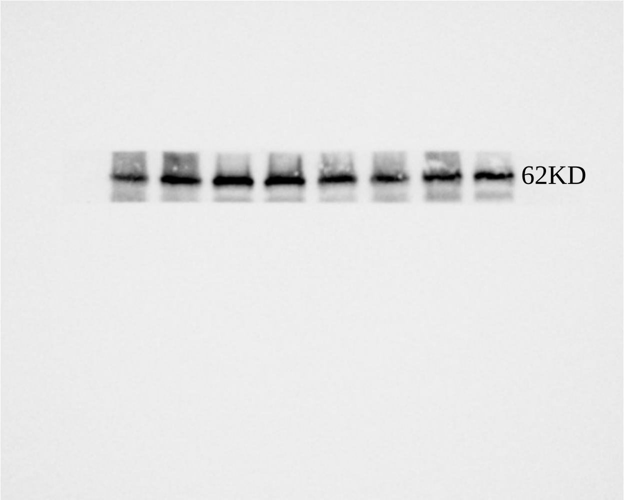

Figure 8C p-JNK 5

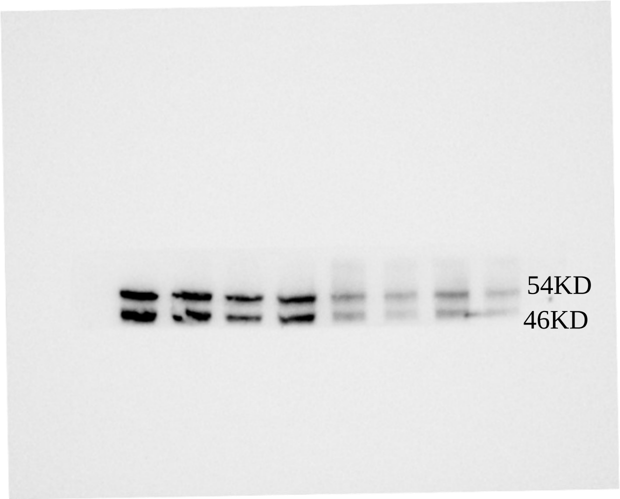

Figure 8C JNK

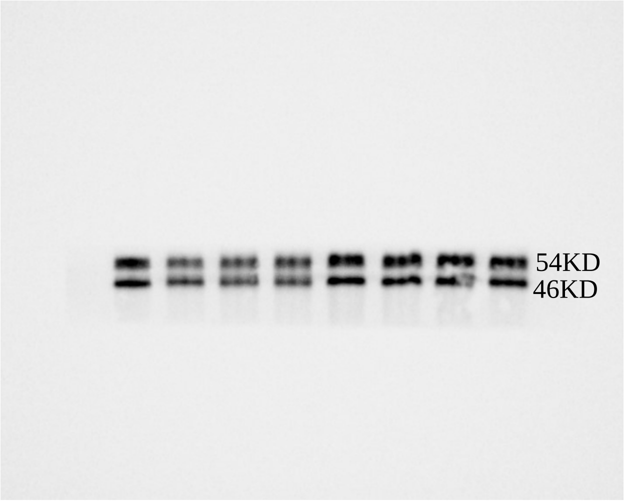

Figure 8C cleaved caspase-3

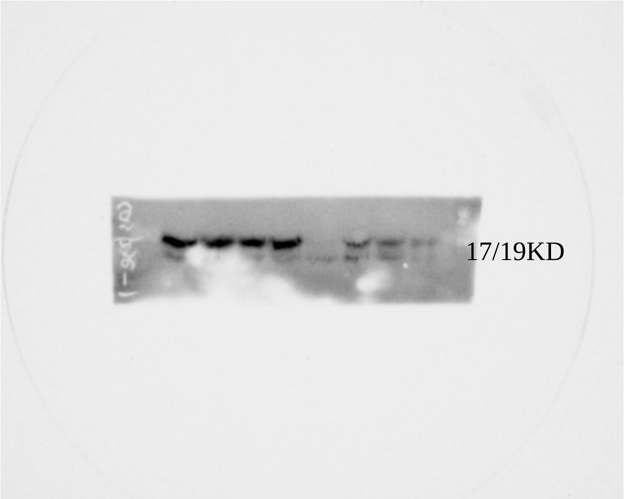

Figure 8C β-actin

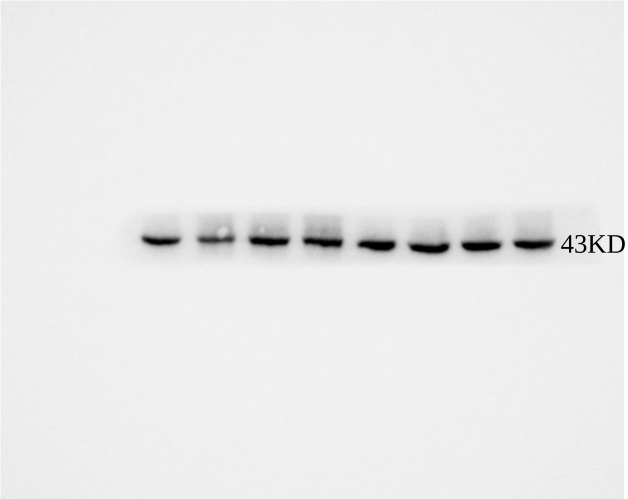

S1D IRG1

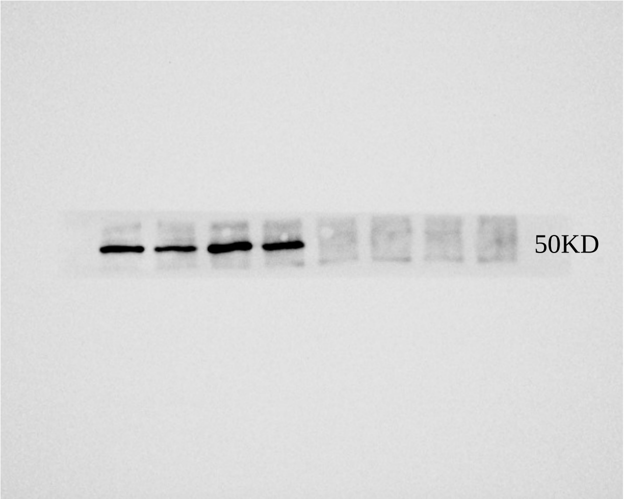

S1D β-actin

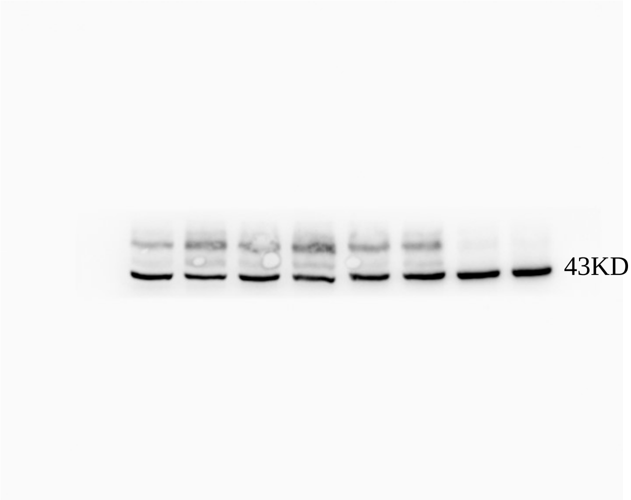

S3A p-AMPK

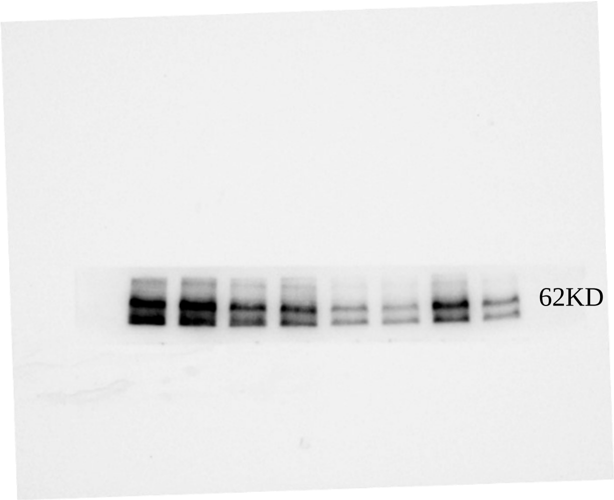

S3A AMPK

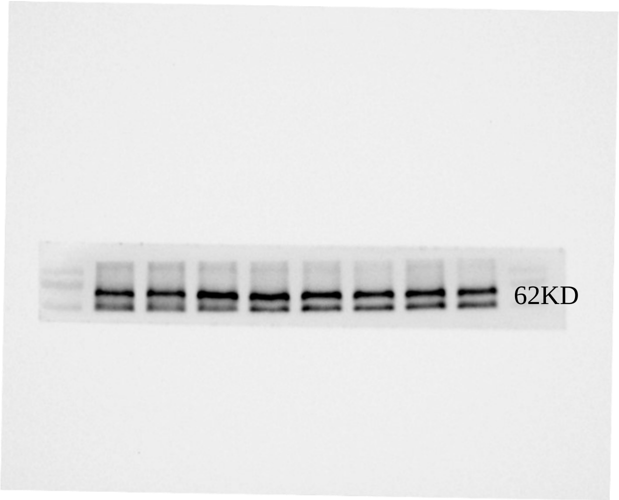

S3A p-JNK

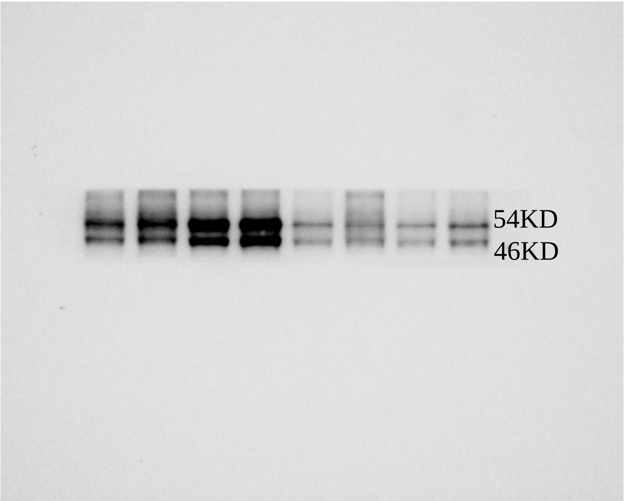

S3A JNK

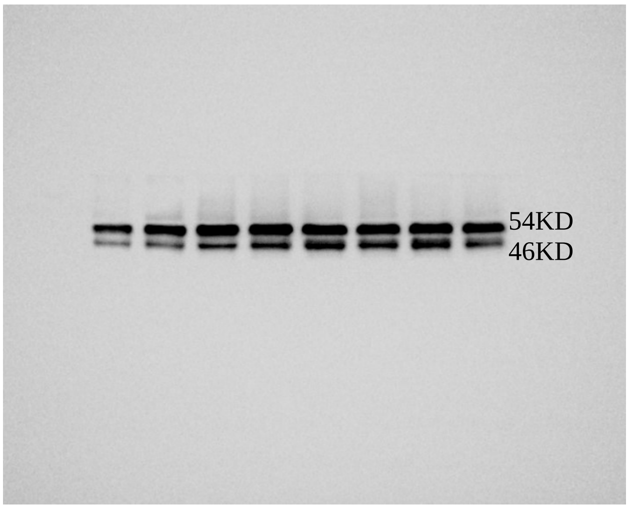

S3A Cleaved caspase-3

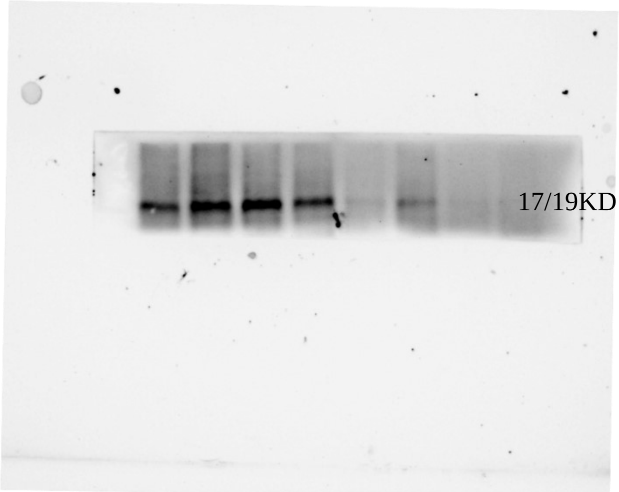

S3A β-actin

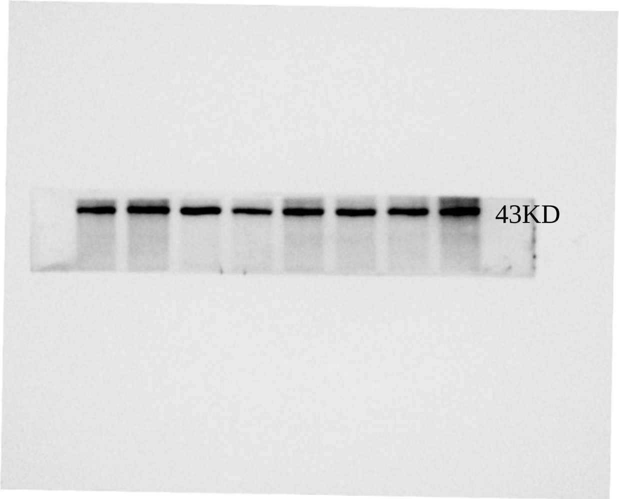

S4A p-JNK

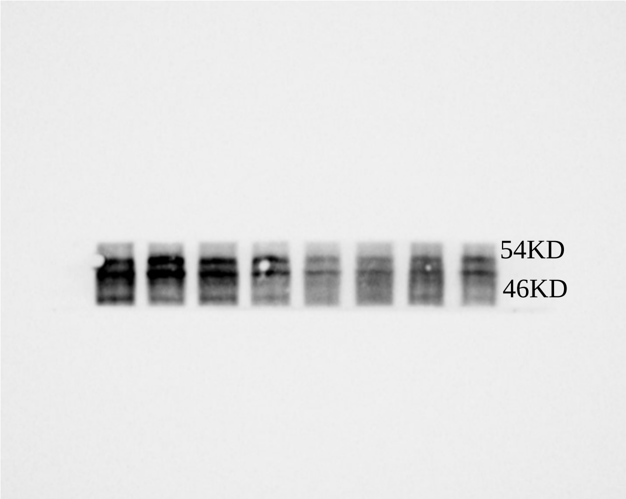

S4A JNK

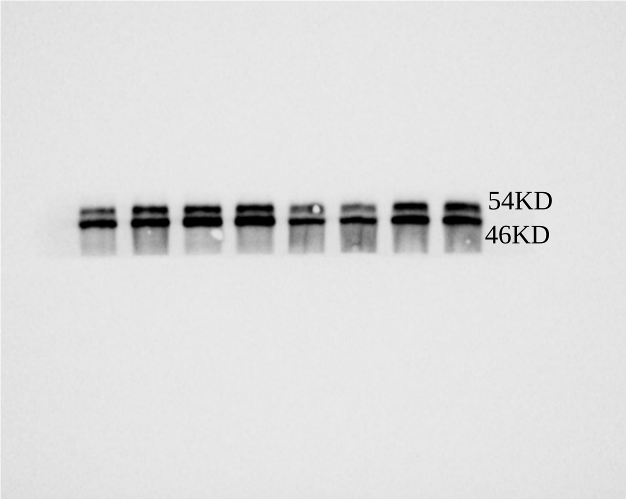

S4A Ceaved caspase-3

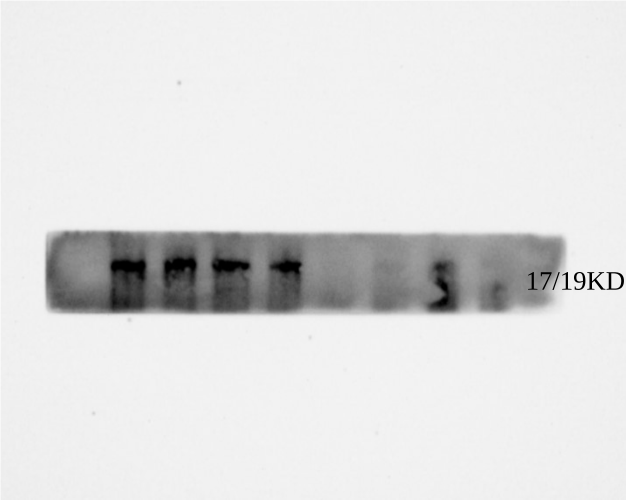

S4A β-actin

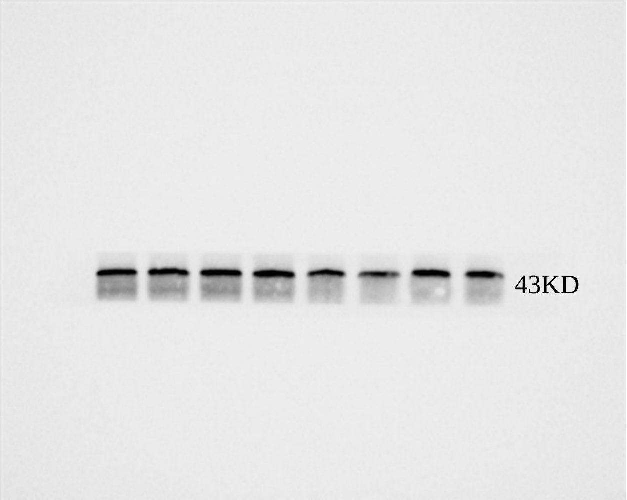

S6C Nrf2

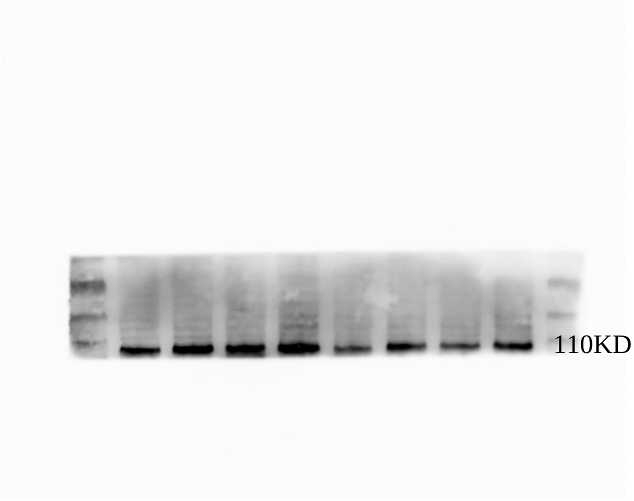

S6C β-actin

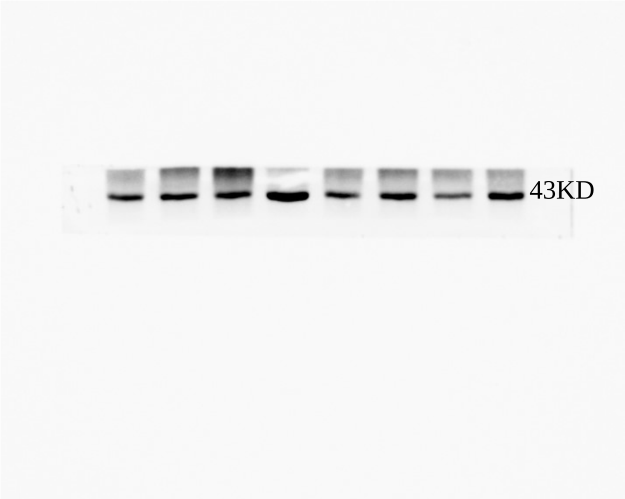

S7B p-AMPK

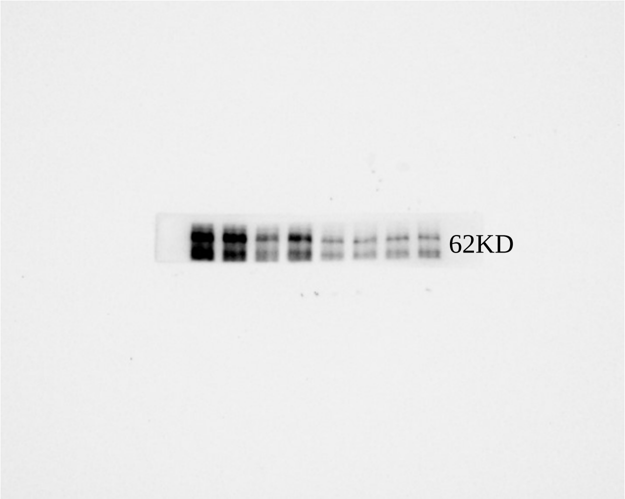

S7B AMPK

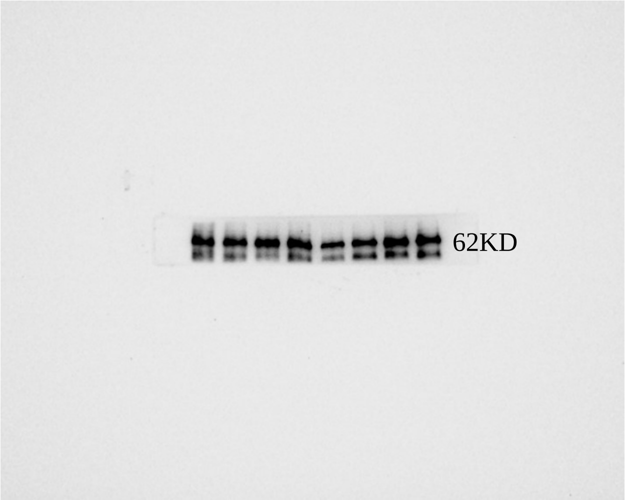

S7B p-JNK

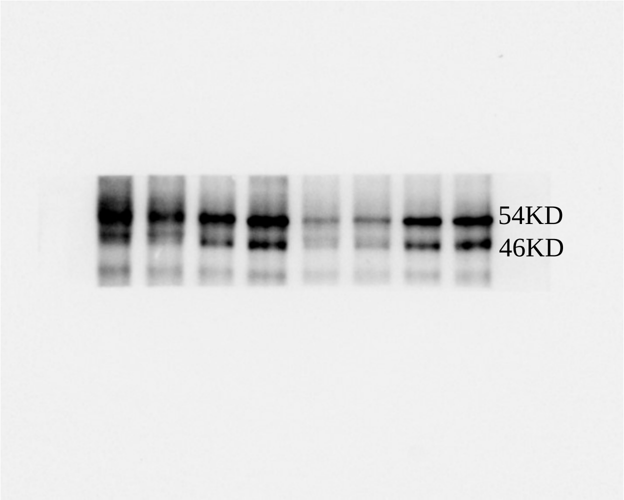

S7B JNK

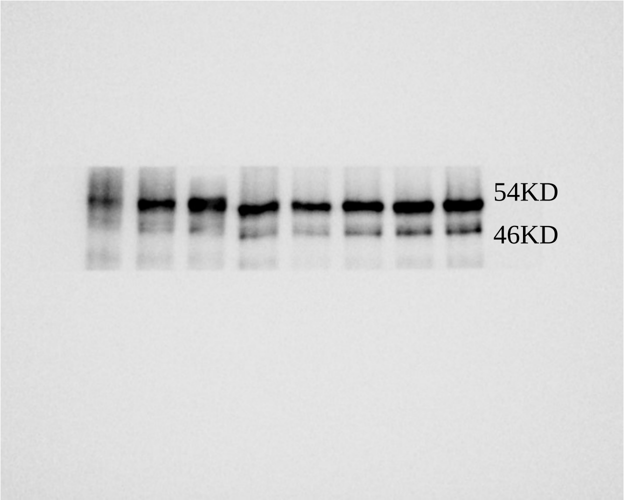

S7B Cleaved caspase-3

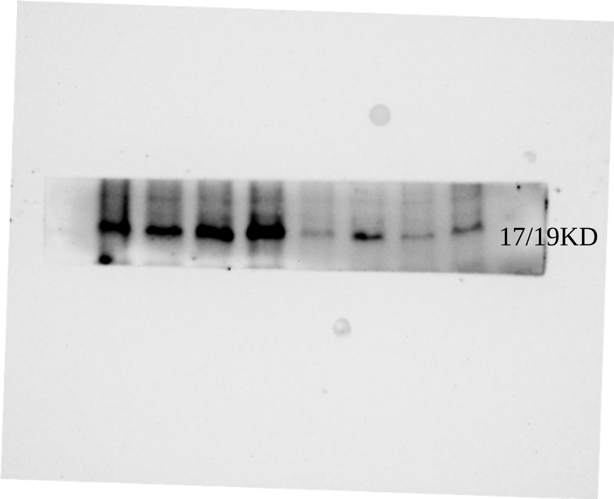

S7B β-actin

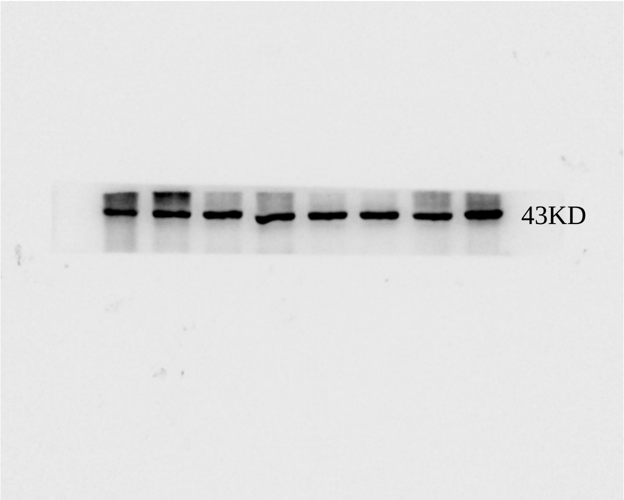

43KD
